# Diversity of major urinary proteins (MUPs) in wild house mice

**DOI:** 10.1038/srep38378

**Published:** 2016-12-06

**Authors:** Michaela Thoß, Viktoria Enk, Hans Yu, Ingrid Miller, Kenneth C. Luzynski, Boglarka Balint, Steve Smith, Ebrahim Razzazi-Fazeli, Dustin J. Penn

**Affiliations:** 1 Konrad Lorenz Institute of Ethology, Department of Integrative Biology and Evolution, University of Veterinary Medicine Vienna, Vienna, Austria; 2 Vetcore Facility for Research, Proteomics Unit, University of Veterinary Medicine Vienna, Vienna, Austria; 3 Institute of Medical Biochemistry, Department of Biomedical Sciences, University of Veterinary Medicine Vienna, Vienna, Austria

## Abstract

Major urinary proteins (MUPs) are often suggested to be highly polymorphic, and thereby provide unique chemical signatures used for individual and genetic kin recognition; however, studies on MUP variability have been lacking. We surveyed populations of wild house mice (*Mus musculus musculus*), and examined variation of MUP genes and proteins. We sequenced several *Mup* genes (9 to 11 loci) and unexpectedly found no inter-individual variation. We also found that microsatellite markers inside the MUP cluster show remarkably low levels of allelic diversity, and significantly lower than the diversity of markers flanking the cluster or other markers in the genome. We found low individual variation in the number and types of MUP proteins using a shotgun proteomic approach, even among mice with variable MUP electrophoretic profiles. We identified gel bands and spots using high-resolution mass spectrometry and discovered that gel-based methods do not separate MUP proteins, and therefore do not provide measures of MUP diversity, as generally assumed. The low diversity and high homology of *Mup* genes are likely maintained by purifying selection and gene conversion, and our results indicate that the type of selection on MUPs and their adaptive functions need to be re-evaluated.

House mice (*Mus musculus*) excrete large quantities of major urinary proteins (MUPs) in their urine, and one function of these proteins is to bind and transport hydrophobic ligands, including volatile pheromones^1^. MUPs are often suggested to be highly polymorphic (high individual variation), and provide distinctive olfactory cues that mediate individual recognition, genetic kin recognition, and inbreeding avoidance (barcode hypothesis)^2^,^3^,^4^,^5^,^6^. However, almost nothing is known about the variation of MUPs at the genetic or protein level, or how such diversity might be maintained by natural selection. In house mice, MUPs are encoded by 21 paralogous genes (and at least 20 pseudogenes) inside a 2 Mb cluster on chromosome 4^7^,^8^. *Mup* genes show very high sequence similarity among loci^8^,^9^, which makes it difficult to understand how these genes could also be highly polymorphic.

Our first aim was to test whether *Mup* genes are highly polymorphic in populations of wild house mice (*Mus musculus musculus*). Because *Mup* loci are so homologous, it is impossible to design locus-specific primers, but on the other hand, a single primer can be used to simultaneously amplify and evaluate polymorphism of multiple *Mup* loci. We directly assessed *Mup* genomic sequence variation with primers targeting exon 2 of several paralogous *Mup* genes. We also assessed genetic diversity of the MUP cluster using a panel of 10 microsatellite markers located inside the cluster, which we compared with 18 markers flanking the cluster, and 9 additional markers on other chromosomes^10^. If *Mup* genes are highly polymorphic, then genetic markers inside the cluster should show elevated levels of allelic diversity compared to other markers flanking the cluster and markers on other chromosomes. Subsequent comparison between sequencing and microsatellite data helped determine whether the pattern of polymorphism in our sampled populations is consistent across genotyping methods.

Our second aim was to test whether MUP proteins show high levels of individual variation in wild mice. Previous claims that MUPs are highly polymorphic were based on measurements of individual MUP profiles using IEF gels, and these studies reported that wild house mice express individually unique MUP profiles or ‘barcodes’^2^,^11^ with 3 to 14 different bands (often referred to as ‘isoforms’) per individual^11^,^12^,^13^. We recently investigated this hypothesis with much larger sample sizes, and we found less individual variability in MUP profiles than expected, i.e., MUP profiles were not individually unique and most (88%) individuals had identical major bands^14^. We also compared individual variation and consistency in MUP profiles for the first time, and we found individual MUP profiles were less consistent than expected, i.e., 71% of individuals did not show consistent profiles^14^. Thus, the inter-individual variation in MUP profiles is potentially explained by within-individual dynamics in MUP expression. As we emphasized, however, IEF and other electrophoretic gels do not necessarily provide measures of variation of MUP proteins. MUP studies have long assumed that different MUPs can be separated by high-resolution techniques, such as isoelectric focusing (IEF) using gels in narrow pH ranges^2^,^11^,^13^, i.e., it has been assumed that different IEF bands represent different MUP proteins, and the number and position of IEF bands provides a measure of MUP protein diversity^2^,^11^,^13^,^15^. Surprisingly, these assumptions have never been validated to our knowledge. Therefore, we analysed variation in MUP proteins in wild mice with gel-based (IEF and two dimensional electrophoresis (2D-PAGE)) and gel-free bottom-up shotgun (Iontrap and QTOF mass spectrometry) proteomic approaches. We explain why our results have important implications for efforts to understand the evolution and functions of MUPs.

## Results

### Genetic diversity of MUPs

Direct Sanger sequencing of PCR amplicons revealed mixed *Mup* sequence reads of 160 bp (after primer removal) of DNA sequence including the full exon 2 of the *Mup* gene region. Unexpectedly, the *Mup* sequence reads consisted of presumably just two distinct variants of mouse *Mup* genes after phase discrimination. Every individual displayed an identical *Mup* sequence read profile (i.e., containing identical ambiguous bases at 15 polymorphic sites throughout the *Mup* sequence read, see Supplemental Fig. S1). The two phased reads did not exactly match any published *Mup* sequence read, but were most similar to *Mup*7 (4 bp difference) and to a non-expressed *Mup* variant (2 bp difference), respectively. These two *Mup* variants are located at the very edges of either end of the central region of the MUP cluster. It should be noted however that, due to the lack of published *Mup* sequences for wild mice and the fact that our primers likely amplify multiple *Mup* genes, the exact location and distribution of our amplified sequences within the MUP cluster is largely unknown. Nevertheless, we estimate that we amplified between 9 and 11 different loci (see Methods), and our results clearly show low *Mup* sequence variation throughout the cluster and among individuals.

### Genetic diversity of MUP-linked microsatellites

**Figure 1:**
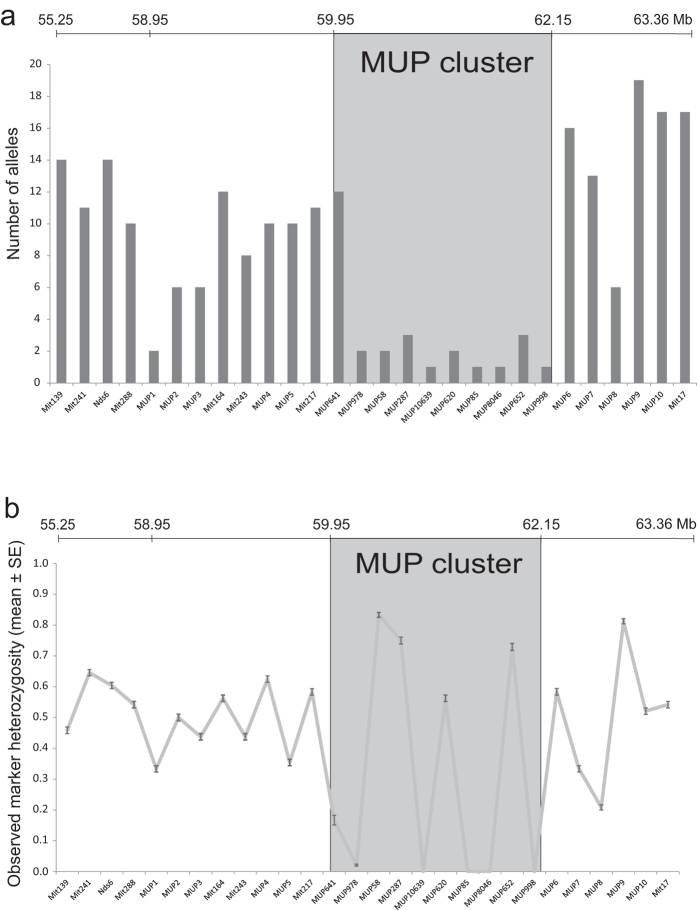
Comparison of (**a**) number of microsatellite alleles and (**b**) observed heterozygosity at microsatellite markers inside (grey box) and outside the MUP cluster (see Supplemental Table S7 for details). Numbers above the graphs indicate genomic position on chromosome 4.

**Figure 2:**
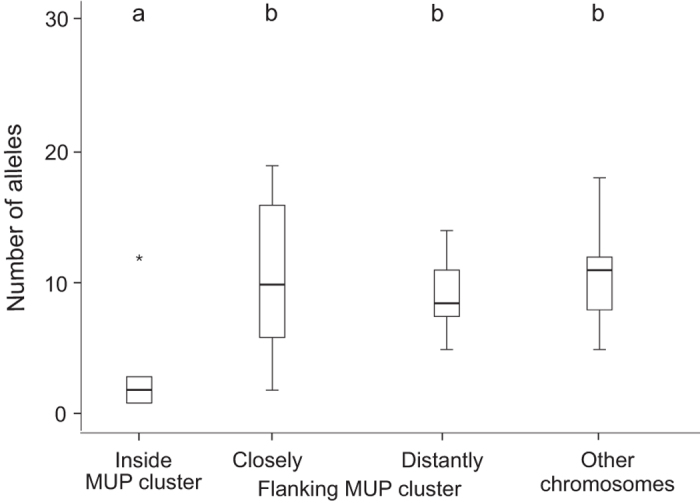
Number of microsatellite alleles at four marker panels (inside and outside the MUP cluster as well as markers on other chromosomes, see Methods and Supplemental Table S7 for details). Different letters above the boxplots indicate statistically significant differences.

**Figure 3:**
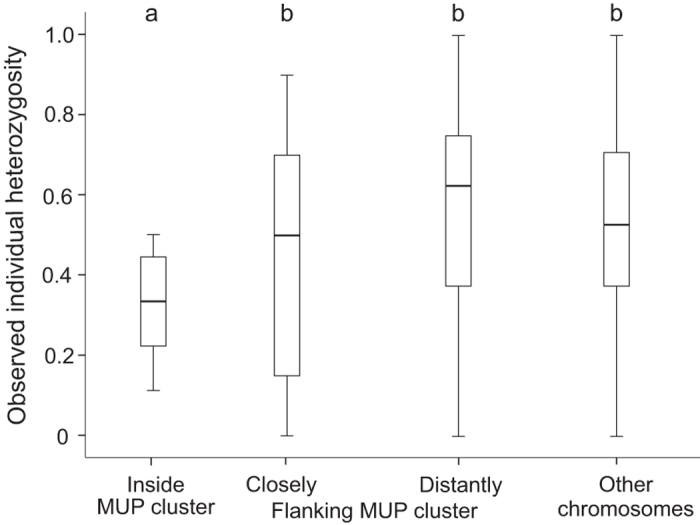
Observed individual heterozygosity at four marker panels (inside and outside the MUP cluster as well as markers on other chromosomes, see Methods and Supplemental Table S7 for details). Different letters above the boxplots indicate statistically significant differences.

We found that genetic markers inside the MUP cluster were not highly polymorphic, and on the contrary, they were less polymorphic than markers outside of the cluster: Allelic diversity and heterozygosity of microsatellites located inside the MUP cluster were significantly lower than markers (closely and distantly) flanking the MUP cluster and markers on other chromosomes (number of alleles, Kruskal Wallis test, N = 48, χ^2^ = 19.11, p = 0.001; posthoc: all χ^2^ ≥ 9.03, all p ≥ 0.0025, Figs 1a and 2; heterozygosity, Kruskal Wallis test, N = 48, χ^2^ = 21.71, p < 0.001, posthoc: all χ^2^ ≥ 29.18, all p < 0.001, Figs 1b and 3). Variance in the number of alleles per locus did not differ between the marker panels (Squared ranks test for equality of variances, χ^2^ = 4.93, p = 0.29). Variance in observed heterozygosity differed between the four marker panels (χ^2^ = 29.18, p < 0.001), however, variance did not differ between the distantly flanking MUP markers and the markers on other chromosomes (pairwise comparisons: distantly flanking MUP markers vs. markers on other chromosomes: p = 0.3; all other comparisons: p ≤ 0.002). We found that microsatellite markers located inside the MUP cluster showed either very low or very high levels of observed heterozygosity (Fig. 1b). To explain this unexpected finding, we considered the possibility that the apparent heterozygous markers were artefacts from amplifying two separate (duplicated) loci. We examined the offspring genotypes produced by parents bred in our colony that were both heterozygous for the same two alleles, and compared the actual versus expected (Mendelian) genotype frequencies (using 9 markers inside and 10 markers closely flanking the MUP cluster). We found that heterozygous parents produced a highly significant excess of heterozygous genotypes for markers within the MUP cluster (χ^2^ = 71.2, df = 2, p < 0.001), whereas for markers flanking the cluster, offspring genotypic frequencies did not differ from Mendelian expectations (χ^2^ = 3.52, df = 2, p = 0.17, Supplemental Fig. S2). We can rule out abortional selection against homozygotes at these markers, as mice produced normal litter sizes (range: 3 to 9 pups, mean: 5 pups). These findings are consistent with the hypothesis that highly heterozygous markers inside the cluster were artefacts from amplifying duplications.

### Individual diversity of MUP proteins

**Figure 4:**
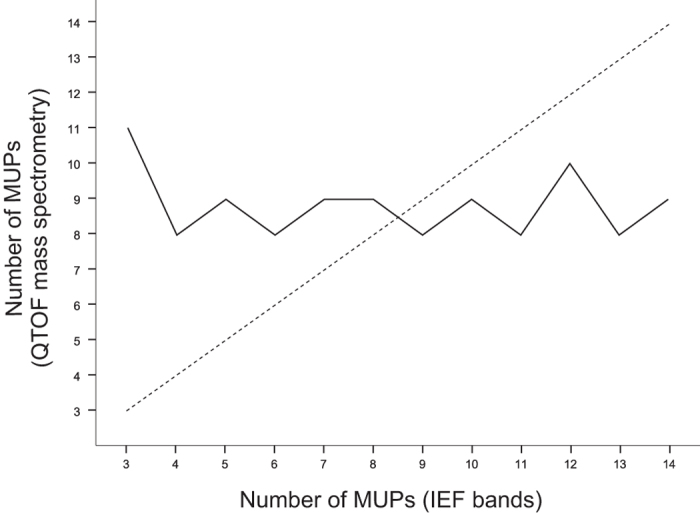
Correlation of number of MUP bands obtained using isoelectric focusing (IEF bands) and number of MUPs detected in a shotgun approach with protein identification using QTOF mass spectrometry (Method C). Dashed line indicates the expected relationship between the number of IEF bands and MUP proteins.

We conducted proteomic analyses to assess inter-individual variation of MUP proteins, and we compared results from different techniques. We analysed variation in individual mice showing the complete range of MUP electrophoretic (IEF) profiles (the standard technique for measuring MUP variation), expressing from 3 to 14 bands per individual (details in ref. 14), and used a shotgun approach coupled with high-resolution quadrupole time-of-flight mass spectrometer (QTOF-MS) to determine how this diversity correlates with the actual number of MUP proteins. Despite selecting mice with the maximum diversity in MUP IEF bands, the mice expressed approximately 9 MUP proteins per individual on average (range: 8 to 11 MUPs, technical repeatability: 98%; Fig. 4). Moreover, the similarity of MUP proteins between individuals (mean: 0.7 ± 0.1), measured with mass spectrophotometry, did not correlate with IEF similarity (mean: 0.7 ± 0.1; Spearman’s rank correlation, ρ = −0.07, p = 0.40, Supplemental Fig. S3). These findings show that wild mice express low variation in the number and type of MUP proteins in urine, even among mice with a complete range of diversity in IEF gel profiles.

### Identification of MUP proteins

We aimed to confirm that IEF and 2D gels separate MUP proteins, as generally assumed. First, we conducted an in-solution digestion of urine samples and identified five different MUP proteins per individual and six MUP proteins in a sample of four pooled individuals (Supplemental Table S1). Subsequently, both samples were run on narrow-range IEF gels and we identified proteins in individual bands using ion trap mass spectrometry (IT-MS). We analysed six IEF bands from the individual sample (Supplemental Fig. S4), and found four different MUP proteins; three IEF bands contained more than one MUP protein and two MUP proteins occurred in two and three bands, respectively (Supplemental Table S2A). We confirmed these findings by analysing five corresponding IEF bands from the pooled sample (Supplemental Fig. S5). Bands from the pooled sample contained MUPs similar to those found in the individual sample (Supplemental Table S2B) and, again, bands contained more than one MUP protein and two MUPs occurred in more than one band. These results indicate that IEF gels do not separate MUP proteins, and that the same MUP protein often occurs in different IEF bands. Since we found IEF bands contain more than one MUP protein, we separated MUP IEF bands by protein size in a second dimension (2D-PAGE) and continued identifying spots using IT-MS. In total, we analysed 15 spots from the individual sample (Supplemental Fig. S4) and 17 spots from the pooled sample (Supplemental Fig. S5). Again, we found that 2D-PAGE spots also contained more than one MUP protein, and the same MUP proteins occurred in different spots (Supplemental Tables S3 and S4). We conducted several additional analyses to identify individual MUP proteins in our samples using QTOF-MS, which confirm that 2D-PAGE spots contained more than one MUP protein (up to 12) and the same MUP proteins occurred in different spots (see Supplemental Results; Supplemental Figs S6–S8; Supplemental Tables S5 and S6). Thus, these findings indicate that gel-based methods do not separate MUP proteins.

## Discussion

We found surprisingly low levels of inter-individual variation in MUP genes and proteins in populations of wild house mice, contrary to suggestions that both are highly polymorphic^2^,^3^,^4^,^5^,^6^. Through DNA sequencing, we found no individual variation, and every individual carried the same two distinct *Mup* variants. Our *in silico* PCR simulations suggested that we would amplify at least 11 separate genomic regions of both central and peripheral *Mups* (and sequencing of control line DNA indicated that our primers should detect nine described *Mup* loci and an additional three undescribed base changes). This lack of inter-individual sequence variation strongly indicates conserved and invariant alleles within the *Mup* gene cluster, contrary to what has been previously assumed.

We also found remarkably low levels of allelic diversity of microsatellite loci throughout the MUP cluster, and less diversity than compared with markers outside the cluster. We analysed the diversity of 10 microsatellite markers located inside the MUP cluster, and found that all markers (except one) had remarkably low levels of allelic diversity (mean: three alleles), and they were significantly less polymorphic than 18 other markers flanking the cluster and 9 other arbitrarily chosen markers located on other chromosomes (mean: 11 and 13 alleles respectively). Only one marker located on the centromeric edge of the MUP cluster showed allelic diversity comparable to that of outside markers, though heterozygosity at this marker was very low. This marker does not reflect overall MUP diversity inside the cluster, and interestingly, it is located close to *Mup4*, which is expressed in lacrimal glands and olfactory mucosa^16^, and may have different functions than urinary MUPs. The only other study utilizing microsatellite markers to infer MUP diversity to our knowledge suggested that MUPs are highly polymorphic and provide the genetic basis for kin recognition and inbreeding avoidance^4^. We derived one panel of microsatellite markers from this previous study, which are surprisingly located up to 7.2 MB away from the MUP cluster, and we used these markers to assess variation at microsatellites ‘distantly flanking MUP cluster’. We found that the allelic diversity and heterozygosity of these ‘distantly flanking MUP cluster’ markers were significantly higher than for markers inside the MUP cluster, which indicates that they should not be used as surrogates for MUP alleles or measuring MUP diversity.

We found that markers inside the MUP cluster showed either very low or very high individual heterozygosity. To explain this finding, we considered the possibility that heterozygous markers within the MUP cluster were artefacts from amplifying different (duplicated) loci, fixed for different alleles. We tested whether the heterozygous markers inside the MUP cluster showed Mendelian assortment, and we found a striking deficiency of offspring with homozygous genotypes. We can rule out segregation distortion and *in-utero* selection against MUP homozygotes. Thus, the markers that are apparently heterozygous are most likely duplications, which is consistent with recent evidence of copy number variation (CNVs) in *Mup* genes in wild house mice^17^. This finding suggests that heterozygosity and allelic diversity inside the MUP cluster might be even lower than what we have estimated.

Our proteomic results also indicated that wild mice express low inter-individual variation in the number of urinary MUP proteins – despite that we analysed mice expressing the complete variation in MUP IEF profiles in these mouse populations. We found that the number of MUP proteins can be reliably inferred using a shotgun approach coupled with high-resolution mass spectrometry, a bottom-up strategy that can detect single amino acid differences between highly similar proteotypic peptides, in contrast to other MS-based strategies^18^. Using this shotgun proteomic approach, we found that less variation in the number (between 8 and 11 MUP proteins per individual) and types (on average 73% similarity) of MUPs proteins among individuals than expected. MUP diversity in our study is an estimate from 8 different populations, and individual MUP variation *within* populations is likely to be even lower.

To our surprise, we also found that the standard gel-based techniques (IEF and 2D gels) used to measure MUP variation do not measure the number or identity of MUP proteins. A low resolution IT-MS instrument identified up to four MUP proteins per 2D-PAGE spot (co-migration) and the same MUP occurred in several spots (proteoform ref. 19). High-resolution mass spectrometry (QTOF-MS) showed that there were also different MUPs within single 2D-PAGE spots (up to 12 proteins) and, again, the same MUP occurred in several different spots. These findings show that gel-based separation techniques do not provide accurate methods to quantify the number of excreted MUP proteins in urine samples or to identify individual MUP proteins. Some previous proteomics studies have found similar examples of co-migration of different proteins, and separation of the same protein into different bands and spots (proteoforms)^20^,^21^,^22^. Narrow and ultra-narrow range pH gradients have been recommended to separate co-migrating proteins^23^,^24^, however, ultra-narrow pH ranges did not resolve the high number of very similar MUP proteins in our study. Previous studies on MUPs have suggested that under native conditions, ligand binding (volatile pheromones ref. 1) or post-translational modifications, such as glycosylation^25^, may change the charge and hence electrophoretic migration behaviour of individual MUPs. Such effects could explain why we found that the same MUP protein can occur at different locations in gels, along the pH and molecular weight (MW) gradients. Thus, MUPs do not show migration behaviour as predicted from the (theoretical) MW and charge, which precludes MUP identification based on positions in the gel. Also, MUPs appear to differentially interact with SDS during 2D-PAGE, which results in two spot rows and further complicates 2D-PAGE spot pattern interpretation. Although measures from gel-based methods do not correspond to variation in MUP proteins measured by gel-free techniques, they might provide information about MUP molecules, such as variation in ligands or post-translational modifications (PTM), and therefore, these techniques might still be complementary. Single amino acid differences may only be detectable with high-resolution MS techniques, but gel-based separation techniques might help detect PTMs or other modifications, if they change the electrophoretic migration behaviour. The new proteomic technologies open opportunities for quantifying MUP variation, but additional methods may be needed to explain functional diversity of MUPs.

A recent study published while our manuscript was in review also investigated genetic and proteomic variation in MUPs of wild house mice (*Mus musculus domesticus*)^26^. It was suggested that individuality arises through a combination of genetic variation in amino acid coding sites and differential transcription of central *Mup* genes across individuals (‘combinatorial diversity’ hypothesis). The genetic analyses detected some sequence variation in *Mup* genes and promoters, but no measures of individual variation were reported and no comparisons with other loci were made. The results confirmed that different *Mup* loci are highly homologous^9^ and provided evidence that they are under purifying selection, which is consistent with our findings. The proteomic analysis examined variation and consistency in individual urine protein profiles, as we previously conducted^14^, though they used a top-down, ESI/MS approach, in which MUPs were classified by the mass of intact proteins. This method is not comparable to our bottom-up, peptide-based approach to identification, and it is more similar to IPG approaches, which attempt to categorize MUPs based on charge/pI of intact protein-ligand complexes (see above). There were no tests for unique individual MUP profiles, and all individual mice expressed the eight proteins selected for the analyses, consistent with our findings (but contrary to the barcode hypothesis). The relative intensities of the MS peaks of these eight proteins were used to assess quantitative variation and consistency between two samples per individual collected over 0–30 days in the laboratory. The mice showed more inter- than intra-individual variation in peak intensities of these proteins, indicating consistent individual profiles^14^. However, further analyses are needed to confirm protein identification, evaluate variation and consistency across all *Mup* loci, and over a longer time and across different conditions. MUP production is dynamically regulated with changes in age^27^, health^28^,^29^, diet^30^, and social status^31^,^32^, and we recently found that MUP proteins are differentially regulated when males are placed in competitive social conditions^33^. Thus, MUP expression profiles are phenotypically plastic, rather than constitutive (developmentally fixed), and studies are needed to investigate how variation and consistency in MUP expression influences individual *odor*, especially under more natural conditions.

In conclusion, our results provide several new perspectives on the evolution and diversity of MUPs, and the methods used to study this interesting family of genes and proteins. First, we found no evidence to support proposals that *Mup* genes are highly polymorphic^11^,^34^, and on the contrary, *Mup* genes showed no individual variation whatsoever. Moreover, microsatellite markers throughout the MUP cluster showed remarkably low levels of allelic diversity, and significantly lower diversity than markers located outside the MUP cluster. It is important to note that we investigated a larger number of mice than previous studies (48 mice from eight different populations), and that these mice otherwise showed comparable levels of heterozygosity to other outbred populations and no evidence of inbreeding^35^. Second, our proteomic analyses using gel-free methods showed that wild mice express surprisingly low individual variation in MUP proteins, even for mice that show high variation in MUP gel profiles, which is consistent with our genetic analyses. Third, we attempted to explain the disparity between gel-based and gel-free proteomic methods, and unexpectedly we found that gel-based methods do not separate MUP proteins. Our findings indicate the electrophoretic methods used for studying MUPs for the past 80 years^2^,^15^ – to infer similarity^2^, diversity^11^,^13^,^15^ and other variations in MUPs^30^,^36^, including hormonal regulation^37^,^38^ and epigenetic control^39^, need to be re-evaluated. Gel-based methods provided the basis for the barcode hypothesis and claims that MUPs are highly polymorphic^2^,^4^ – and therefore, the hypothesis that MUPs mediate individual odour and genetic kin recognition (inbreeding avoidance)^2^,^3^,^4^,^6^,^13^ also needs to be re-evaluated. The low individual variation of *Mup* genes and linked microsatellites suggest that these genes are evolving under purifying selection^26^, perhaps through a selective sweep in this large region^40^, and the high homology of different *Mup* loci may be generated by concerted evolution (gene conversion)^41^,^42^. It is unclear why selection favours low individual variation and high homology of MUP loci, and studies are needed to investigate possible effects on individual survival^43^, as well as reproductive success (through chemical communication).

## Methods

### Genetic diversity of MUPs

#### Subjects

To investigate genetic variation, 48 mature wild house mice (*Mus musculus musculus*) were trapped in barns, stables or farms at eight localities in Austria. For details see ref. 14. To investigate the complete lack of homozygotes at markers inside the MUP cluster, we compared the genotypes of parents (N = 64) and their offspring (75 offspring from 14 single-sired litters, mean litter size: 5, range: 3–9 pups). For further details see Supplemental Methods. The experimental procedures were conducted in accordance with ethical standards and guidelines in the care and use of experimental animals and approved by the Ethical and Animal Welfare Commission of the University of Veterinary Medicine Vienna and the Austrian Federal Ministry of Science and Research (Permit No. BMWF-68.205/0225-II/3b/2012 and 02/08/97/2013).

#### Sanger sequencing

To estimate *Mup* sequence variation, we designed PCR primers matching exon 2 of multiple *Mup* genes based on homology to the published *Mup4* gene using the Primer-BLAST tool of the NCBI database (Forward primer: 5′-GTA TCC CTG CTC CTT CTC CCT-3′, reverse primer: 5′-CTC AGC TAG GAG CAT CTC ACT T-3′). Primers were additionally tested for sequence homology to other genomic regions using the UCSC *in silico* PCR tool (http://genome.ucsc.edu/cgi-bin/hgPcr). *In silico* PCR analysis suggested that four separate variants (each placed within the peripheral MUP cluster) would be amplified from a direct match to our primer set. A further seven variants of central *Mups* varied by just a single base in the forward priming site and were also expected to amplify under our PCR conditions. Additionally, we expected to capture variation at an even larger portion of *Mup* genes for which no published sequence information currently exists. To validate our *in silico* results, we amplified DNA from a control laboratory strain (C57BL/6 J, JAX Labs, Bar Harbor, Maine), and confirmed that we could assign eight of the 11 *Mup* sequences that were predicted to be amplified by *in silico* PCR. Four variants, representing three pseudogenes *Mup* alleles predicted to amplify from the control strain (*Mup*6ps, *Mup*10a-ps, *Mup*19ps), were not detected with our primer set (Supplemental Fig. S1). We detected four additional variants in the control line not predicted from our *in silico* PCR, one matching the sequence for *Mup*8 and three others not previously recorded.

PCR reactions were carried out in 25 μl reactions containing 1 × PCR buffer (Solis Biodyne, Estonia), 2 mM MgCl_2_, 200 μM of each dNTP, 0.5 U Firepol Taq polymerase (Solis Biodyne, Estonia), 0.2 μM of each primer and 20–40 ng of template DNA. PCR cycling was performed on a Life Technologies Pro-flex Thermocycler under the following conditions: denaturation at 94 °C for 5 min followed by 35 cycles of 94 °C for 30 s, 58 °C for 30 s, and 72 °C for 60 s with a final extension of 7 min at 72 °C. Prior to sequencing, products were purified with Exonuclease I and Shrimp Alkaline Phosphatase to remove residual primers and nucleotides. Cycle sequencing reactions were performed in forward and reverse directions using the Applied Biosystems Big Dye Terminator version 3.1 ready reaction cycle sequencing kit and were resolved on an Applied Biosystems 3130xl Genetic Analyzer (ThermoFisher Scientific). Secondary peak calling and alignment of sequences was performed in CLC workbench (v. 7.6.4) with 0.3 set as the fraction of the maximum peak height for calling a minor peak. All calls were checked by eye before phased alleles were resolved using the program PHASE^44^ implemented within DNAsp^45^.

#### Microsatellite markers

Due to the high sequence similarity of different *Mup* loci, it is impossible to develop locus-specific primers, and therefore we utilized microsatellite markers to estimate and compare genetic diversity at microsatellite markers inside and outside the MUP cluster^40^. DNA was extracted from ear punches and other tissue samples using a proteinase K/isopropanol protocol^46^. 48 adults were genotyped at 39 microsatellite loci in different multiplex runs. Ten microsatellite markers were located inside the MUP cluster (‘inside MUP cluster’ panel), ten were closely flanking the cluster (‘closely flanking MUP cluster’ panel; up to 1 MB downstream or up to 1 MB upstream of the MUP cluster), and eight additional markers were more distantly flanking the cluster (‘distantly flanking MUP cluster’ panel; between 0.6 and 7.2 MB up- and downstream ref. 4). We genotyped nine additional markers on eight different chromosomes (‘other chromosomes’ panel ref. 14) to compare genetic diversity inside and near the MUP cluster with other parts of the genome. For details see Supplemental Table S7.

For multiplex PCR, amplification mixes were subjected to a denaturation step at 95 °C for 15 min followed by 35 cycles at 94 °C for 30 s, respective annealing temperature for 90 s and 72 °C for 60 s, followed by an elongation step at 72 °C for 10 min. For a detailed list of annealing temperatures for each marker see Supplemental Table S7. Amplification products were analysed using an automated sequencer (Applied Biosystems 3130xl Genetic Analyzer, ThermoFisher Scientific). Allele scoring was conducted using GeneMarker V2.6.0 software and allele sizes were determined with a homemade size standard.

### Diversity of MUP proteins

#### Subjects

From the mice trapped for the genetic survey (see above), urine samples of 12 males were selected based on the number of IEF gel bands and they were used for MUP protein identification by shotgun proteomics (see Method A below). To obtain samples for gel-based experiments, urine was repeatedly collected from adult first generation (F1) offspring of wild-caught house mice (*Mus musculus musculus*, trapped in Vienna, Austria) using metabolic cages (Tecniplast, Germany). Urine was collected and pooled from a single, adult male (‘individual urine sample’). Urine samples of four adult, unrelated, same age males with different IEF patterns were combined to obtain the ‘pooled urine sample’.

#### MUP identification

Gel-based proteomic approaches were initially conducted using an ion-trap instrument, and after purchasing new triple TOF instrument, experiments were performed using high-resolution MS. High-resolution MS does not require gel-based separation before peptide measurement and thus, is better suited for analysis of multiple highly homologous proteins such as MUPs. For a typical chromatogram of mouse urine measured with QTOF-MS and an overlay of TIC chromatograms of 26 nanoLC-MS runs see Supplemental Fig. S9. More information about the technical repeatability of our methods for MUP quantification can also found in another recent paper^33^.

#### Method A: MUP identification by shotgun proteomics

Mass spectrometric protein identification of shotgun samples was based on a nano high performance liquid chromatography electrospray quadrupole time of flight mass spectrometer system (nanoHPLC-ESI-QTOF-MS, TripleTOF 5600+, Sciex, USA). Due to high MUP homology, data were recorded from m/z =  250 to 1500. Data acquisition and interpretation was performed using Analyst 1.7, ProteinPilot 5.0 (both Sciex, USA) and Chromeleon (Dionex, The Netherlands). Using the Triple TOF strategy, all variable modifications were searched at once rather than independently during protein identification with ProteinPilot. For more details see Supplemental Methods.

#### Method B: MUP identification from one-dimensional isoelectric focusing gels (immobilized pH gradient gels, IPGs)

IEF under native conditions was performed as previously described^14^, and variation in MUP proteins of individual mice expressing 3 to 14 bands per individual was analysed. Contrary to what has been suggested^26^, the less conspicuous or “minor” bands in IEF gels are not artefacts generated by storage, i.e., minor bands appear in fresh and frozen urine samples (unpublished data). IEF bands of interest were excised from the strip and subjected to a modified sample preparation protocol for MS identification^47^. Mass spectrometric protein identification of IEF bands was conducted using a nanoHPLC-ESI iontrap mass spectrometer (nanoHPLC-ESI-IT-MS, HCT esquire, Bruker, Germany). Data acquisition and interpretation were done using HyStar Software 3.2 (Bruker Daltonics, Germany) combining Esquire Control 6.1 (Bruker Daltonics, Germany) and the Chromeleon DCMS link (Dionex, The Netherlands), as well as ProteinScape 2.0 (Bruker Daltonics, Germany). Using the Bruker Ion Trap instrument, we performed a fragmentation based on data-dependent acquisition strategy by using a unique peptide list. For more details see Supplemental Methods.

#### Method C: MUP identification after two-dimensional gel electrophoresis (2D-PAGE)

In this study, 2D-PAGE was conducted using a combination of native IEF as described in Method B (see above) and sodium dodecyl sulphate polyacrylamide gel electrophoresis (SDS-PAGE). For SDS-PAGE, separation gels (140 × 140 × 1.5 mm) were prepared with a 10–20% T gradient in the upper half and 20% homogenous gel in the lower half, and a stacking gel (5% T) was polymerized on top of the separation gel. After silver staining, individual spots were excised and further sample preparation was performed according to Method B with adaptations for trypsin digestion at pH 8.5. Peptide separation and protein identification was performed with nanoHPLC-ESI-IT-MS as described in Method B adapted for trypsin. Spots from 2D gels were also analysed using nanoHPLC-ESI-QTOF-MS (see Method A). For more details see Supplemental Methods.

#### IEF gel and MUP protein similarity

IEF similarity was calculated as described in ref. 14. Similarly, to obtain MUP similarity, proteins detected using shotgun proteomics (Method A) were aligned for all samples and we calculated the Manhattan distance between two samples (e.g., scoring 1 for each match (protein present or absent in both samples) and 0 for each mismatch (protein present or absent in one of the samples but not in the other) and averaged over all comparisons. This matching coefficient ranges from 0 (0% MUP similarity) to 1 (100% MUP similarity or identical MUP composition) and was square-root transformed for statistical analysis.

#### Statistical analyses

Average values are reported as mean ± standard deviation. We tested for differences in mean number of alleles per locus and observed heterozygosity between the four panels of microsatellite markers using Kruskal-Wallis tests and subsequent pairwise comparisons with Bonferroni correction. We used squared ranks test and subsequent pairwise comparisons with Bonferroni correction to investigate differences in variance of number of alleles per locus and observed heterozygosity between the four panels of microsatellite markers. We used Chi-square tests to compare expected and observed offspring allele frequencies at microsatellite markers inside and closely flanking the MUP cluster. We used Spearman’s rank correlation to test for a correlation of number of IEF bands and number of MUPs identified using QTOF-MS; and to correlate IEF similarity and MUP similarity. Identifications were performed using a MUP database (in-house Mascot server), and were considered statistically significant if p < 0.01. We used a 1% False Discovery Rate (FDR) and confidence threshold = 0.05. In our study we used unique peptides as a parameter to validate identification of MUPs (unlike other proteomics studies, we could not use a minimum number of two identified peptides per protein because MUPs are so highly homologous and often differ only by a single unique peptide). To confidently identify proteins, a decoy database was created from reversed database entries and the FDR was calculated from performing a search against this database. Statistical analyses were performed using SPSS 20.

### Data Accessibility

The full data set will be made available upon publication (protein sequences will be submitted to the PRIDE repository, and gene sequences will be submitted to Genbank).

## Additional Information

**How to cite this article**: Thoß, M. *et al*. Diversity of major urinary proteins (MUPs) in wild house mice. *Sci. Rep.* **6**, 38378; doi: 10.1038/srep38378 (2016).

**Publisher's note:** Springer Nature remains neutral with regard to jurisdictional claims in published maps and institutional affiliations.

## Supplementary Material

Supplementary Information
