# Supplementary material for: Diversity of major urinary proteins (MUPs) in wild house mice
Source: Sci Rep. 2016 Dec 6;6:38378. doi: 10.1038/srep38378 (PMC5138617; doi:10.1038/srep38378)
Supplement: Supplementary Information [file srep38378-s1.pdf]

Diversity of major urinary proteins (MUPs) in wild house mice

Thoß, Michaela[1], Enk, Viktoria[2], Yu, Hans[2§], Miller, Ingrid[3], Luzynski, Kenneth C[1], Balint, Boglarka[1], Smith, Steve[1], Razzazi-Fazeli, Ebrahim[2], Penn, Dustin J[* 1]

[1] Konrad Lorenz Institute of Ethology, Department of Integrative Biology and Evolution, University of Veterinary Medicine Vienna, Vienna, Austria

[2] Vetcore Facility for Research, Proteomics Unit, University of Veterinary Medicine Vienna, Vienna, Austria

[3] Institute of Medical Biochemistry, Department of Biomedical Sciences, University of Veterinary Medicine Vienna, Vienna, Austria

[§] present address:

Institute of Biotechnology in Animal Production, IFA Tulln, Tulln, Austria and Institute of Animal Breeding and Genetics, University of Veterinary Medicine Vienna, Vienna, Austria

[*]Corresponding author: Dustin Penn, dustin.penn@vetmeduni.ac.at

Supplemental Materials

Supplemental Methods

MUP Genetics

We confirmed our *in silico* predictions with a C57BL/6 reference genome, and found that most variations in MUP sequences were detected (see Supplemental Fig. S1)[1].

Deficiency of homozygotes

We found that markers inside the MUP cluster showed either very low or very high heterozygosity. To explain the complete lack of homozygotes at some markers, we used parents and their offspring from semi-natural population enclosures. In short, adult mice (N = 64) were released into indoor enclosures (3.4 x 4 m each) containing wooden bedding (ABEDD, Austria), plastic nest boxes, a water station, wood wool, and paper towels as nesting material. New born were removed and tissue samples were stored at -20 °C for paternity analysis using 10 to 12 microsatellite markers[2]. Paternity was determined by complete exclusion using the program CERVUS 3.0.3[3]. Parents and their offspring were additionally genotyped at nine microsatellite markers inside the MUP cluster and ten markers closely flanking the MUP cluster (see above). We compared expected (Mendelian inheritance, AA: 0.25, AB: 0.50, BB: 0.25) and actual offspring genotype frequencies at markers at which both parents were heterozygous for the same two alleles (AB; inside MUP cluster: range: 3-4 markers, average 3.5 markers; closely flanking cluster: range: 0-9 markers, average 3 markers).

MUP Proteomics

Sample preparation and data acquisition

Method A: MUP identification by shotgun proteomics

All proteomics analyses were carried out using LC-MS grade solvents and chemicals. Protein concentration was measured using standard Bradford assay[4] and 5 µg were used for tryptic in-solution digestion. TRIS-HCl (50 mM pH 8.0) was added to a volume of 10 µL and urea was added (10 µL, 8 M in 50 mM TRIS-HCl pH 8.0) before reducing with 2 µL dithiothreitol (DTT, 50 mM in 25 mM ammonium bicarbonate (ABC)) for 30 minutes at 60°C (Eppendorf Thermomixer comfort). Reduced proteins were subsequently alkylated using 6 µL 2-Iodoacetamide (IAA, 55 mM in 25 mM ABC) for 45 min. at room temperature in the dark. Excess IAA was reduced by another addition of DTT as described above. For digestion, 10 µL ABC (25 mM) and 100 ng trypsin were added and

incubated at 37°C overnight. The digest was stopped by addition of 20 % trifluoro acetic acid (TFA, 1 µL). Mass spectrometric protein identification of shotgun samples was based on a nano high performance liquid chromatography electrospray quadrupole time of flight mass spectrometer system nanoHPLCESI- QTOF-MS, TripleTOF 5600, Sciex, USA). Peptides were separated with an Ultimate 3000 RSLC using a pre-concentration trap column (PepMap100 C18) and a nano separation column (Acclaim PepMap RSLC 75 µm x 25 cm, nano Viper C18, 2 µm, 100 Å) (Dionex, The Netherlands). Peptides were separated with a gradient from 4 % B (80 % acetonitrile (ACN) with 0.1 % formic acid) to 35 % B in 120 min and then up to 90 % B in 15 min followed by a washing step with 90 % B for 10 min. Mobile Phase A consisted of H2O with 0.1 % formic acid. We performed instrument calibration using a ß-Galactosidase digest (Sciex, USA) before each sample injection. The mass measurement accuracy was <2 ppm RMS, a mass tolerance of 50 ppm was used for auto-calibration of the instrument. Detection was carried out on a high resolution QTOF mass spectrometer online coupled to the LC with an ESI source. Due to high MUP homology, data were recorded from m/z= 250 to 1500 to also cover differences in short terminal fragments for protein differentiation. A Top 25 method was chosen with the following parameters:

Experiment Type	TOF MS1	TOF MS2
Number of Cycles	4728	4728
Polarity	Positive	Positive
Period Cycle Time	3050 ms	3050 ms
Pulser Frequency	14.127 kHz	14.127 kHz
Accumulation Time	250.0 ms	110.0 ms

Collision energy for MS/MS experiments was ramped automatically in order to achieve optimal fragmentation for each precursor mass. For a typical single chromatogram of mouse urine measured using QTOF-MS and an overlay of TIC chromatograms of 26 nanoLC-MS runs see Supplemental Fig. S9.

Method B: MUP identification from one-dimensional isoelectric focusing gels (immobilized pH gradient gels, IPGs)

Isoelectric focusing (IEF) is a gel-based proteomic technique used to separate proteins by differences in their isoelectric point (pI). IEF gels are made using immobilized pH gradient (IPG) polyacrylamide gels. During electrophoresis, proteins are separated based on their relative content of acidic and basic residues and focused into sharp bands (each protein at its specific pI).

IEF under native conditions was performed as in [5]. In short, urine samples were separated on 125 mm long IPG-gels with narrow range pH gradients of 4.2-4.9 as a standard or ultra-narrow range pH 4.3- 4.7 in selected cases, followed by protein detection with Coomassie Brilliant Blue R-250. IEF bands of interest were excised from the strip and subjected to a modified sample preparation protocol for MS identification according to [6]. Generally, for all steps a volume of 100 µL and 25 mM ABC was used for washing. After destaining with ABC100/ethanol 50:50 (v/v), reduction with 10 mM DTT in 25 mM ABC (56°C for 1 h) and alkylation with 55 mM 2-iodoacetamide in 25 mM ABC (25°C for 45 min), bands were washed again and dehydrated with ACN. Trypsin digestion was performed according to [7] with 10 µL of 75 ng/µL trypsin (Promega, USA) in 25 mM ABC for 8 h at 37°C. Digest was stopped with 5 µL of 1 M ammonium hydroxide. Peptides were extracted with 3x 30 µL ACN/H2O/TFA 50/45/5 (v/v), dried and desalted using ZipTips C18 (Millipore, USA) according to the manufacturer's instructions. Desalted peptides were reconstituted in 0.1 % TFA.

Mass spectrometric protein identification of IEF bands was done as described above (see Method A), however, a nano high performance liquid chromatography electrospray iontrap mass spectrometer (nanoHPLC-ESI-IT-MS, HCT esquire, Bruker, Germany) with a different nano separation column (Acclaim PepMap RSLC 75 µm x 50 cm, nano Viper C18, 2 µm, 100 Å) (Dionex, The Netherlands) was used. We performed fragmentation based on a data dependent acquisition strategy by using a unique peptide list.

Method C: MUP identification after two-dimensional gel electrophoresis (2D-PAGE)

In this study, 2D-PAGE was conducted using a combination of native IEF as described in Method B (see above) and sodium dodecyl sulphate polyacrylamide gel electrophoresis (SDS-PAGE), thus separating proteins first by their pI and subsequently by their molecular weight (MW). This setup is different from classical two-dimensional electrophoresis which uses denaturing/reducing IEF in the first dimension. However, we chose this setup to ensure comparability to 1D patterns of Method B. For the first dimensional run, 5 mm strips were cut from narrow range dry plates, rehydrated and proteins (5 µg) separated as described[5]. Post separation, strips were frozen until use. For SDS-PAGE, separation gels (140x140x1.5 mm) were prepared with a 10-20 % T gradient in the upper half and 20 homogenous gel in the lower half, for improved separation of low molecular mass compounds. A stacking gel (5 % T) was polymerized on top of the separation gel, the IEF strip put on it and fixed in place by 1 % agarose. Separation was performed in a Hoefer SE600 vertical electrophoresis chamber (Hoefer Scientific Instruments, San Francisco, CA, USA) and proteins stained with an MS compatible silver stain[8]. Individual spots were excised and destained as described in [9]. Further sample

preparation was performed according to Method B with adaptations for trypsin digestion at pH 8.5. For all washing steps 100 mM ABC was used. Tryptic digest was conducted with 0.2 µg of trypsin (Trypsin Gold, Mass Spectrometry Grade, Promega, USA) in 50 mM ABC and 5 mM calcium dichloride for 8 h at 37°C. Peptide separation and protein identification was performed with nanoHPLC-ESI-IT-MS as described in Method B adapted for trypsin and using a nanoHPLC- ESI-QTOF-MS (see Method A).

MUP identification

In our study, protein identification was based on unique peptides of MUPs. In contrast to other proteomics studies, we could not set a minimum number of two identified peptides per protein because MUPs are so highly homologous and often differ by a single unique peptide only. Identification of MUP proteins was considered significant if one proteotypic peptide was identified. A decoy database was created from reversed database entries and the FDR was calculated from performing a search against this database. A protein confidence threshold of 0.05 was used and the FDR was 1 %.

Database construction

For database searches on an in-house Mascot server 2.3.01 (Matrix Science, UK), a database of isoform-specific unique peptides was created based on UniprotKB/Swiss-Prot, UniprotKB/TrEMBL, NCBInr and a MUP cDNA database[10]. This approach utilized all available MUP sequence information by including both reviewed (independently confirmed MUP sequences) and unreviewed (MUP sequences from computationally generated annotation and/or not independently confirmed) MUP entries. Allowing variable modifications, we were able to choose a peptide for identification. Proteins were identified based on peptide sequences computed from MS/MS spectra and identification was not influenced by any PTMs as the software takes possible PTMs into account.

Search strategies and identification parameters for the Triple TOF data sets

For the Q-TOF instrument, the mass tolerance on the MS level was 0.05 Dalton (=68.6 ppm for ß-Galactosidase digest peptide of 729.37 Da) and the MS/MS tolerance was 10 ppm for protein identifications. Data acquisition and interpretation was performed using Analyst 1.7, ProteinPilot 5.0 (both Sciex, USA) and Chromeleon (Dionex, The Netherlands). Database searches with Protein Pilot 5.0 were conducted using the following search parameters: taxonomy all, global

modifications carbamidomethylation on cysteine; variable modifications: Acetyl (protein N-term), Deamidated (NQ), Gln->pyro-Glu (N-term Q), Oxidation (M); enzyme trypsin or pepsin; one missed cleavages allowed. All variable modifications were searched at once during protein identification with ProteinPilot.

Search strategies and identification parameters for the Ion Trap data sets

For the ion trap instrument, the mass tolerance on the MS level was 0.4 Dalton (=548.4 ppm for ß-Galactosidase digest peptide of 729.37 Da) and the MS/MS tolerance was 400 ppm for identifications. Data acquisition and interpretation were done using HyStar Software 3.2 (Bruker Daltonics, Germany) combining Esquire Control 6.1 (Bruker Daltonics, Germany) and the Chromeleon DCMS link (Dionex, The Netherlands) as well as ProteinScape 2.0 (Bruker Daltonics, Germany) using a unique peptide list.

Supplemental Results

To identify individual MUP proteins in our samples, we conducted several additional analyses using high resolution quadrupole time-of-flight mass spectrometry (QTOF-MS). First, we compared MUP identification performance of both mass spectrometry techniques by selecting seven pairs of 2D-PAGE spots with identical gel position from two separate gels (see Supplemental Figs. S4 and S6, identical numbering). QTOF-MS identified approximately three times more MUP proteins compared to IT-MS (mean: IT-MS: 3.6 ± 1.3; QTOF-MS: 10.1 ± 1.2). The same MUP proteins were identified in 6 out of 7 2D-PAGE spots, only for one spot identified MUP proteins did not overlap (Supplemental table S4). We continued using high-resolution QTOF-MS to identify the proteins in different 2D-PAGE spots (13 spots from the individual sample and 30 spots from the pooled sample; Supplemental Figs. S6 and S7, respectively). The individual sample contained 23 MUPs representing products of 16 (out of 21) known Mup genes. We found 5 to 12 different MUPs per spot (average: 8.0 ± 1.8) and individual MUPs occurred in 1 to 10 different spots (mean: 4.8 ± 2.3, Supplemental table S5). The pooled sample of 4 individuals also contained 23 MUPs representing products of 15 known MUP genes. We found 1 to 8 different MUPs per spot (mean: 5.3 ± 1.8) and individual MUPs occurred in 1 to 24 spots (mean: 7.1 ± 6.8, Supplemental table S6), although an even smaller pH range was used in IEF (pH 4.3 to 4.7). Overall, using an additional second dimensional separation step based on protein size did not increase the resolution of MUPs, although two main rows of spots were detected (at 14 and 18 kDa, respectively). An additional experiment showed that those two spot rows are mainly created by different SDS load on the same MUPs (Supplemental Fig. S8).

Supplemental References

1 Chamero, P. *et al.* Identification of protein pheromones that promote aggressive behaviour. *Nature* **450**, 899-U823, doi:10.1038/nature05997 (2007).

2 Thoß, M., Ilmonen, P., Musolf, K. & Penn, D. J. Major histocompatibility complex heterozygosity enhances reproductive success. *Mol. Ecol.* **20**, 1546-1557, doi:10.1111/j.1365-294X.2011.05009.x (2011).

3 Marshall, T. C., Slate, J., Kruuk, L. E. & Pemberton, J. M. Statistical confidence for likelihood-based paternity inference in natural populations. *Mol. Ecol.* **7**, 639-655, doi:10.1046/j.1365-294x.1998.00374.x (1998).

4 Bradford, M. M. A Rapid and Sensitive Method for the Quantitation of Microgram Quantities of Protein Utilizing the Principle of Protein-Dye Binding. *Anal. Biochem.* **72**, 248-254, doi:10.1016/0003-2697(76)90527-3 (1976).

5 Logan, D. W., Marton, T. F. & Stowers, L. Species Specificity in Major Urinary Proteins by Parallel Evolution. *PLoS ONE* **3**, e3280, doi:10.1371/journal.pone.0003280 (2008).

6 Thoß, M., Luzynski, K., Ante, M., Miller, I. & Penn, D. J. Major urinary protein (MUP) profiles show dynamic changes rather than individual "barcode" signatures. *Front. Ecol. Evol.* **3**, 71, doi:10.3389/fevo.2015.00071 (2015).

7 Jiménez, C. R. & Burlingame, A. L. Ultramicroanalysis of Peptide Profiles in Biological Samples Using MALDI Mass Spectrometry. *Exp. Nephrol.* **6**, 421-428, doi:10.1159/000020551 (1998).

8 Llarrull, L. I., Toth, M., Champion, M. M. & Mobashery, S. Activation of BlaR1 Protein of Methicillin-resistant *Staphylococcus aureus*, Its Proteolytic Processing, and Recovery from Induction of Resistance. *J Biol. Chem.* **286**, 38148-38158, doi:10.1074/jbc.M111.288985 (2011).

9 Miller, I. in *Difference Gel Electrophoresis (DIGE),* Vol. 854 (eds Rainer Cramer & Reiner Westermeier) Ch. 26, 373-396 (Springer Science+Business Media, 2012).

10 Gharahdaghi, F., Weinberg, C. R., Meagher, D. A., Imai, B. S. & Mische, S. M. Mass spectrometric identification of proteins from silver-stained polyacrylamide gel: A method for the removal of silver ions to enhance sensitivity. *Electrophoresis* **20**, 601-605, doi:10.1002/(sici)1522-2683(19990301)20:3<601::aid-elps601>3.3.co;2-y (1999).

```
                        10         20         30         40         50         60         70         80         90        100        110        120        130
               ....|....| ....|....| ....|....| ....|....| ....|....| ....|....| ....|....| ....|....| ....|....| ....|....| ....|....| ....|....| ....|....| ....|
Mm-Mup01       attaatgggg aatggttttc tattctcctg gcctctgaca aaagagaaaa gatagaagaa catggcagca tgagagtttt tgtggagcac atccatgtct tggagaattc cttagctttt aaattccata ctgtg
Mm-Mup02       .......... .g....a.a. ....a..... .......... ......c... .......... .......t.. .....c.... .......... ..t....... .......... .....g.... .......... .....
Mm-Mup18       ........aa .......... .......... .......... .......... .......... .......c.. .......... .......... ...g...... .......... .......... .......... .....
Mm-Mup24       .......... ......a.a. ....a.g... ...a...... .......... .......... .......... .......... ...c...t.. .......... .......... .......c.. .......... t.a..
Mm-Mup21PS     .......... ......a.a. ....a.g... ...a...... .......... .......... .......... .......... ...c...t.. .......... .......... .......c.. .......... t.a..
Mm-Mup03       .......... .....ca.a. ....a..... .......... .g........ .........t a......a.t .t...c.... .c.......a .......... .......... .....t.c.. ...g...... .....
Mm-Mup05PS     .......... ......a.a. ....a.t... ..t....... .......... .......... .......... .....c.... .......... ..t....... .........t .......... ...c...... .....
Mm-Mup12PS     .......... ......a.a. ....a.t... ..t....... .......... .......... .......... .....c.... .......... ..t....... .........t .......... ...c...... .....
Mm-Mup10aPS    .......... ......aca. ....a..... ..t....... .......... c......... .......... .....c.... .......... ..t....... .......... .......... ...c...... .....
Mm-Mup06PS     .......... ......aca. ....a..... ..t....... .......... t......... .......... .....c.... .......... ..t....... .......... .......... ...c...... .....
Mm-Mup19PS     .......... .....ca.a. ....a..... .......... .g........ .......... .......... .....c.... .......... ..t......a .....t.... t......... .......... .....
C57B6/J1       ........rr .r...ywyw. ....m.b... ..yw...... .r...m.... .........w m......n.w .k...s.... .s.s...y.m ..ys...k.. .......w.y .....b.y.. ...b...... y.rk.
Mm-Austria-wild .......... .....ya.a. ....a.y... ..y....... .......... .........w .......r.w .k...c.... .s.......m ..y....... .........y .....y.y.. ...y...... .....
```

Supplemental figure S1. Alignment of reference exon 2 sequences for mouse *Mup* genes expected to be amplified using our primer set together with the actual sequences recorded for control line DNA (C57B6/J1) and for all study samples (MM-Austria-wild). Mouse reference gene names are as per Chamero *et al*. (2007). Dots represent identical bases to the reference sequence for the mouse *Mup 01* gene. Multiple nucleotide peaks detected at a single base position for the control line DNA and study samples are indicated by the corresponding IUPAC ambiguity code.

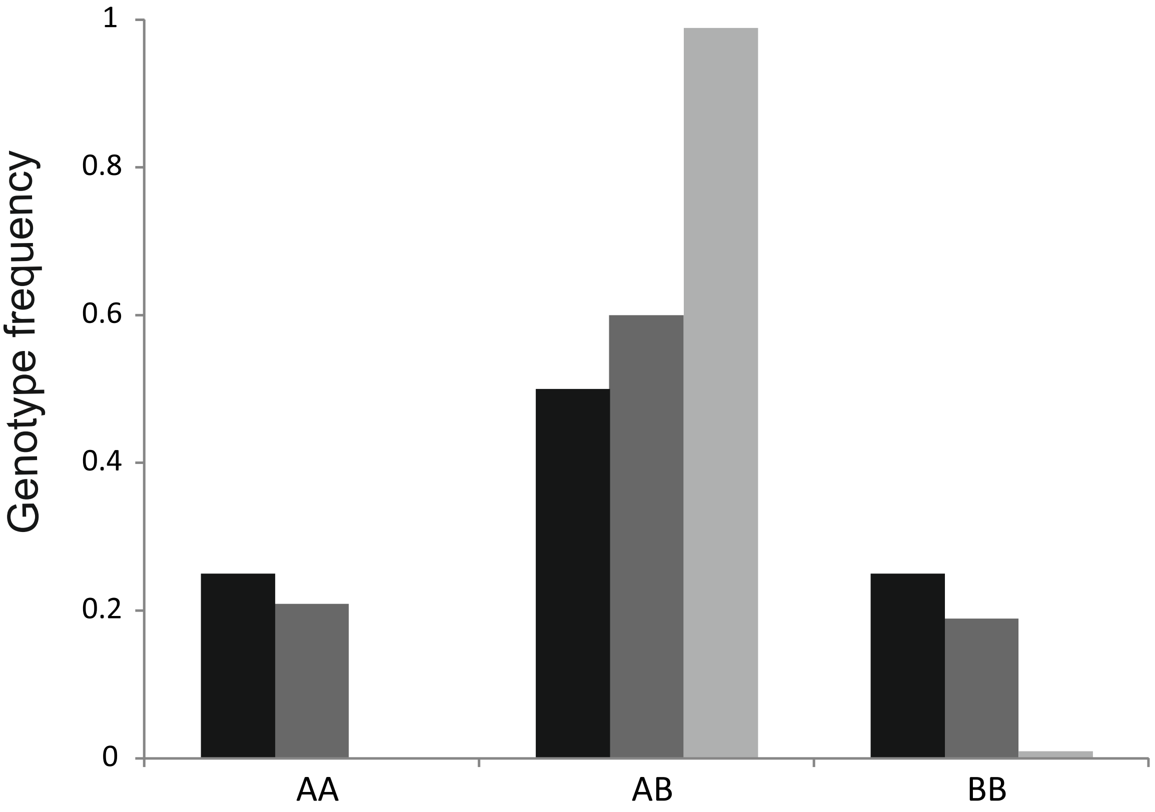

Supplemental figure S2. Deficiency of homozygous offspring genotypes at microsatellite markers inside the MUP cluster. Bar plot shows offspring genotype frequencies at single diallelic loci if parents are heterozygous for AB. Expected offspring genotype frequencies under Mendelian inheritance (black bars), observed offspring genotype frequencies at microsatellite markers closely flanking the MUP cluster (dark grey bars) and observed offspring genotype frequencies at microsatellite markers inside the MUP cluster (light grey bars).

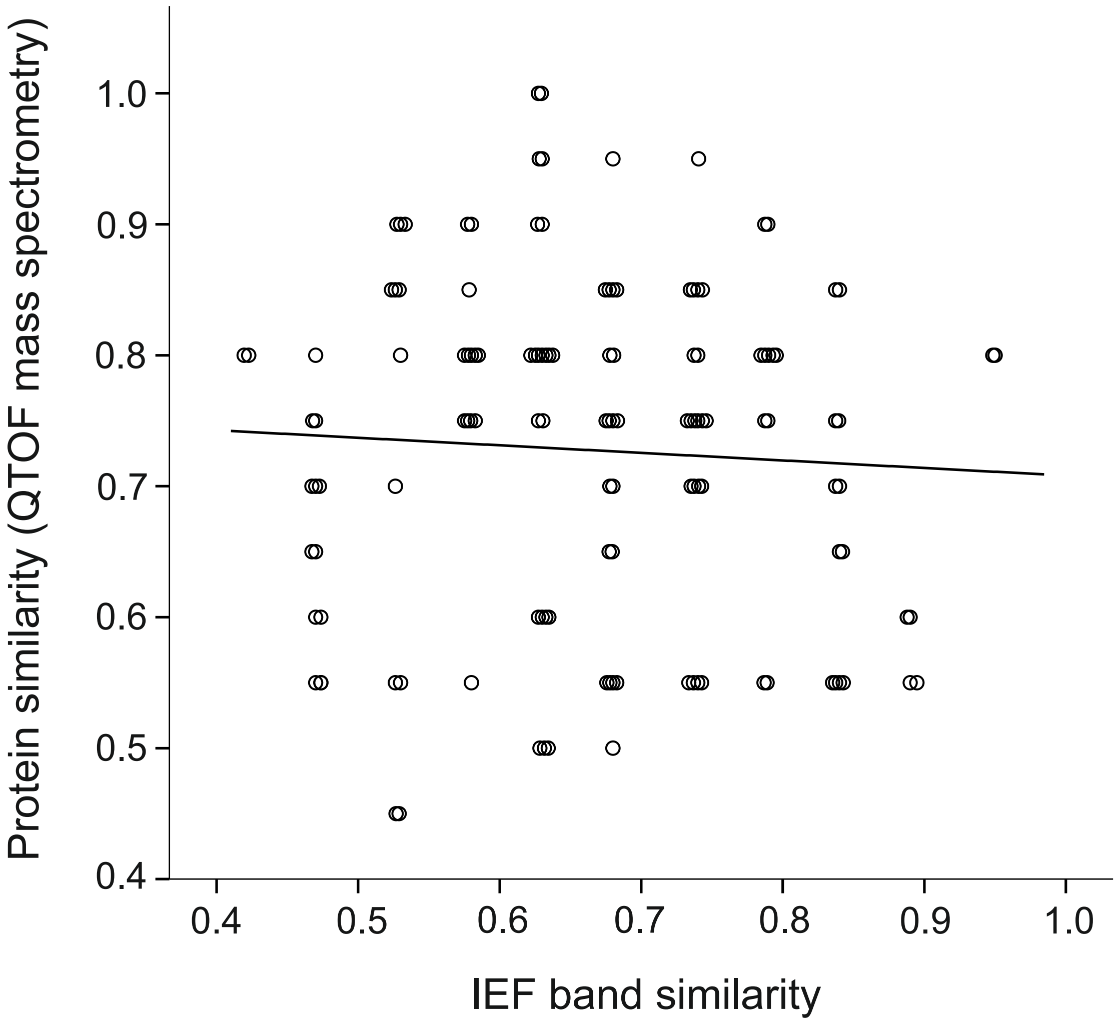

Supplemental figure S3. Correlation of IEF band similarity and MUP similarity (see Methods for details).

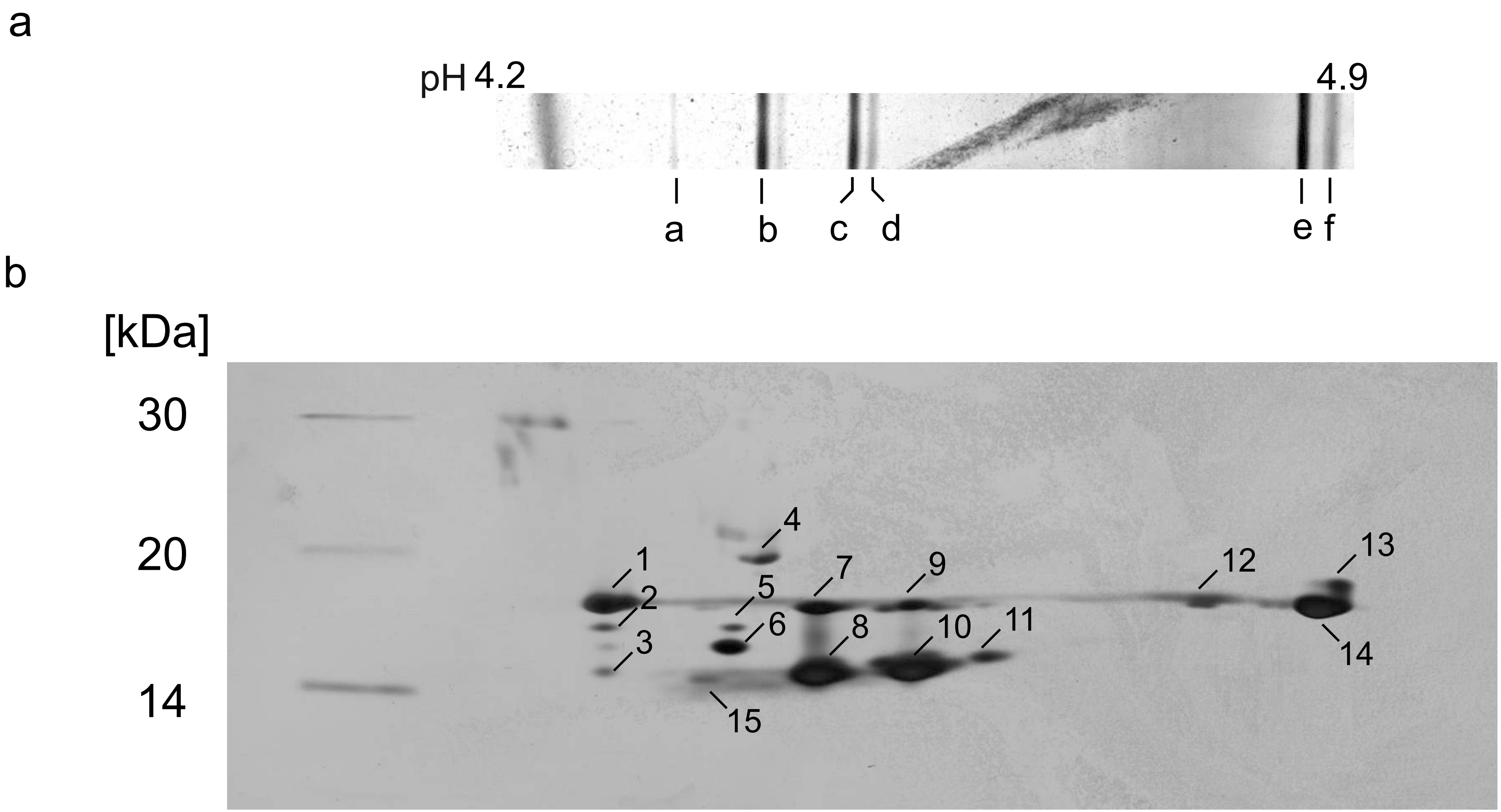

Supplemental figure S4. Individual urine sample separated in two dimensions using (a) isoelectric focusing (IEF) and subsequent (b) SDS-PAGE. In-gel digestion was performed on IEF bands (indicated by small letters, Method B) and 2D-PAGE spots (indicated by numbers, Method C). Protein identification was performed using iontrap mass spectrometry.

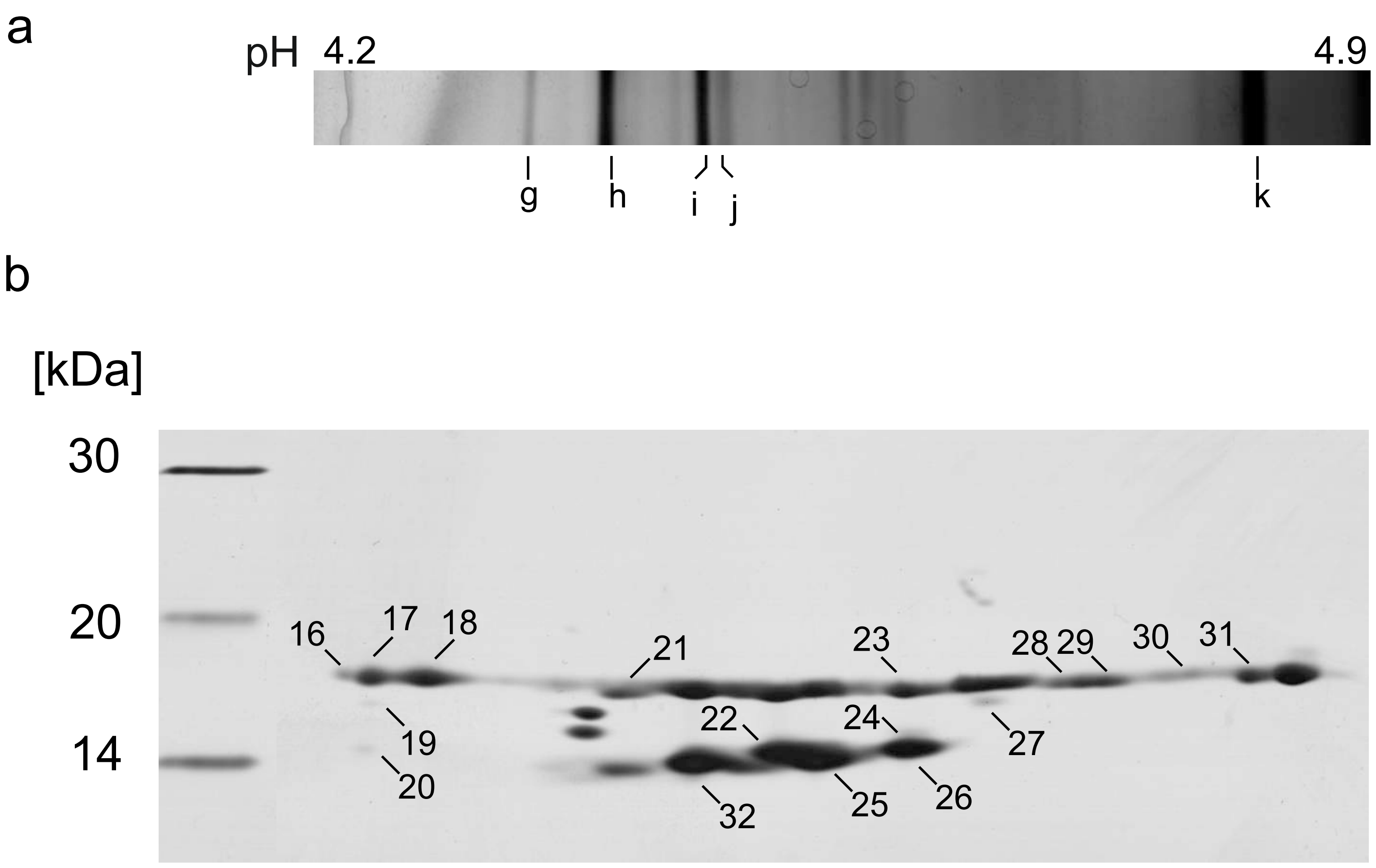

Supplemental figure S5. Pooled urine sample separated in two dimensions using (a) isoelectric focusing (IEF) and subsequent (b) SDS-PAGE. In-gel digestion was performed on IEF bands (indicated by small letters, Method B) and 2D-PAGE spots (indicated by numbers, Method C). Protein identification was performed using iontrap mass spectrometry.

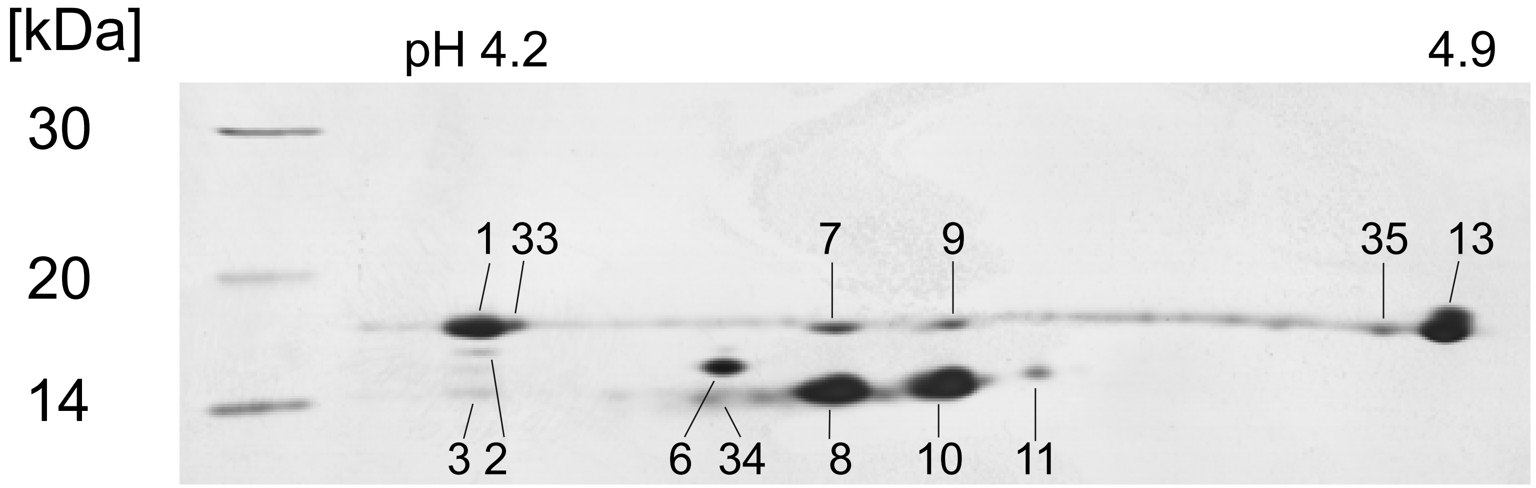

Supplemental figure S6. Individual urine sample separated in two dimensions using isoelectric focusing (IEF) and subsequent SDS-PAGE. In-gel digestion was performed on 2D-PAGE spots (indicated by numbers, Method C). Identical numbers in Supplemental figure S3 and S5 refer to the same SDS-PAGE spots. Protein identification was performed using QTOF mass spectrometry. A comparison of proteins identified with both MS techniques can be found in Supplemental table S4.

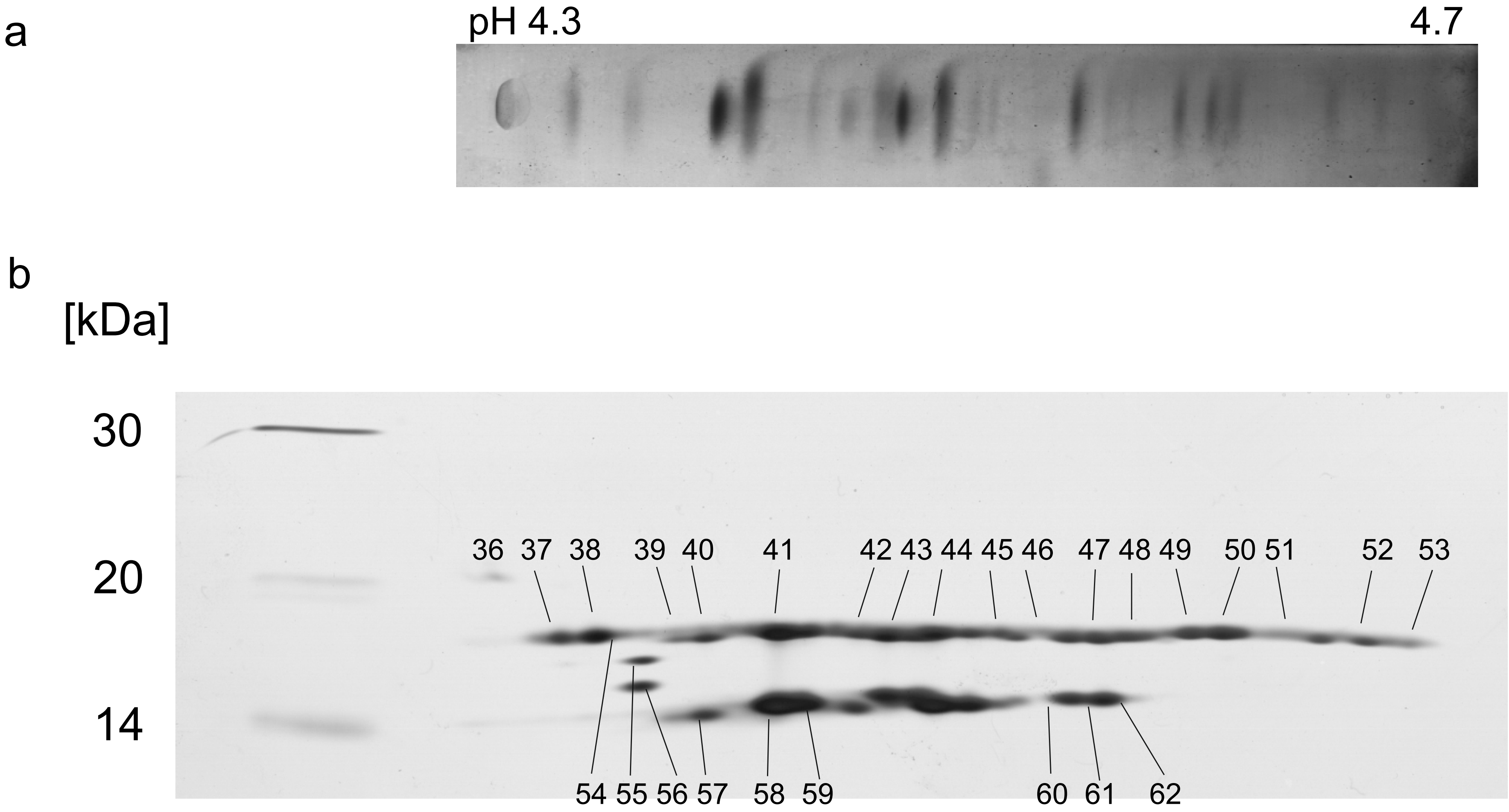

Supplemental figure S7. Pooled urine sample separated in two dimensions using (a) ultra-narrow range isoelectric focusing (IEF, pH 4.3 to 4.7) and subsequent (b) SDS-PAGE. In-gel digestion was performed on 2D-PAGE spots (indicated by numbers, Method C). Protein identification was performed using QTOF mass spectrometry. Note the ultra-narrow pH range (pH 4.3 to 4.7) for IEF.

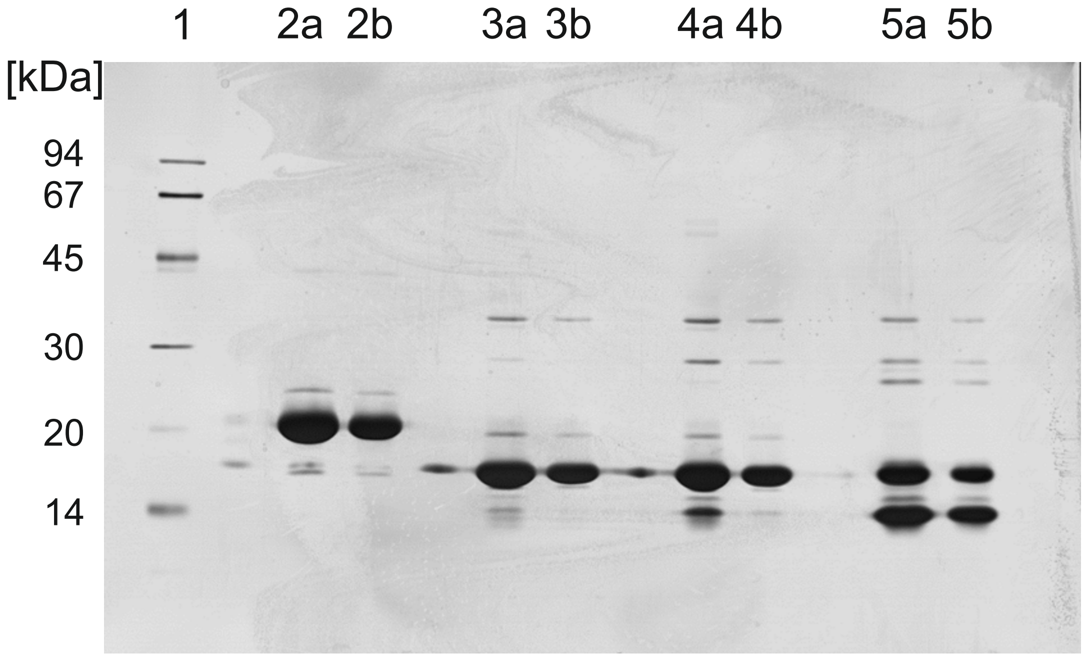

Supplemental figure S8. Influence of urine sample pretreatment on MUP migration behavior on SDS-PAGE. Lane 1: molecular weight marker, lane 2: reduced sample preparation according to Laemmli, lanes 3-5: samples diluted with Laemmli sample buffer, with SDS, but without dithiothreitol (DTT). Lane 3: 3% SDS, lane 4: 0.6% SDS, lane 5: no SDS. 'a' denotes 2.5µg protein, 'b' denotes 1µg protein applied per lane (see Methods). Relative mobility of MUPs is different in reduced (lane 2) and non-reduced states (lanes 3-5). Depending on SDS load, 1-2 main bands of differing intensity appear with an apparent molecular weight of 14 and 17 kD, respectively.

a

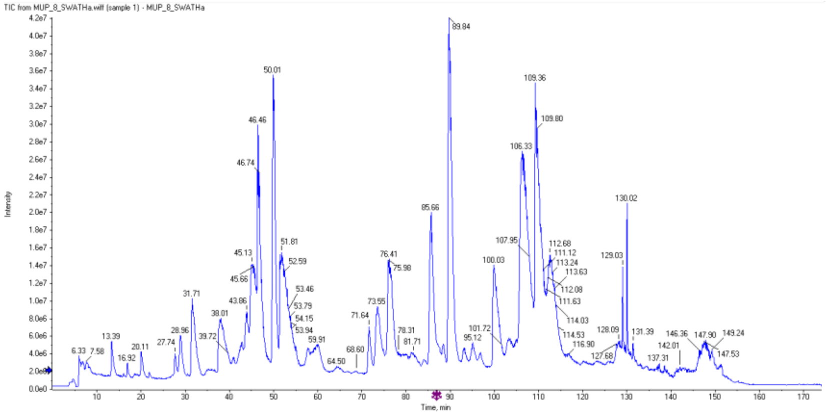

b

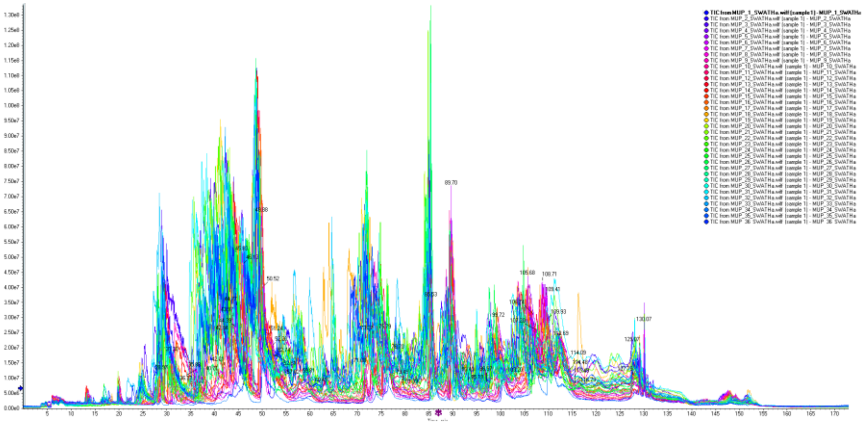

Supplemental figure S9. (a) A chromatogram of a typical MUP sample measured by the QTOF-MS, and (b) An overlay of TIC chromatograms (Q-TOF) from 26 nanoLC-MS runs, which illustrates the reproducibility of our measurements.

Supplemental table S1. Summary of MUPs detected in in-solution digested (A) individual urine sample and (B) pooled urine sample using iontrap mass spectrometry (Method B).

(A) INDIVIDUAL SAMPLE

**Uniprot number**	**Associated *Mup* gene**
tr|P02762	MUP6/8/11
tr|P04938	MUP6/8/11
tr|P11588	MUP1
tr|B5X0G2	MUP17
tr|Q5FW60	MUP20
tr|P04939	MUP3

(B) POOLED SAMPLE

**Uniprot number**	**Associated *Mup* gene**
tr|B5X0G2	MUP17
tr|P02762	MUP6/8/11
tr|P04938	MUP6/8/11
tr|P11588	MUP1
tr|P11589	MUP2
tr|Q5FW60	MUP20
tr|P04939	MUP3

Supplemental table S2. Summary of MUPs detected in in-gel digested bands of (A) individual urine sample and (B) pooled urine sample using iontrap mass spectrometry (Method B). Band labeling as in Supplemental figure 3A and 4A, respectively. N/A = data not available.

(A) INDIVIDUAL SAMPLE

**Band**	**Uniprot number**	**Associated *Mup* gene**
a	tr|Q5FW60	MUP20
b	tr|P02762/P04938, tr|B5X0G2	MUP6/8/11, MUP17
c	tr|P02762/P04938, tr|B5X0G2	MUP6/8/11, MUP17
d	tr|P02762/P04938	MUP6/8/11
e	tr|P02762/P04938, tr|B5X0G2	MUP6/8/11, MUP17
f	tr|P11588	MUP1

(B) POOLED SAMPLE

**Band**	**Corresponding band in individual sample**	**Uniprot number**	**Associated *Mup* gene**
g	a	tr|Q5FW60	MUP20
h	b	tr|P02762/P04938, tr|P11588	MUP6/8/11, MUP1
i	c	tr|P02762/P04938, tr|B5X0G2	MUP6/8/11, MUP17
j	d	-	n/a
k	e	-	n/a

Supplemental table S3. Summary of MUPs identified in 2D-PAGE spots of (A) individual urine sample and (B) pooled urine sample using iontrap mass spectrometry (Method C). 'x' indicates that a specific protein was detected. Labeling of spots as in Supplemental figures 3B and 4B, respectively. No proteins were detected in Spot 2.

(A) INDIVIDUAL SAMPLE

		Spot number														
**Uniprot number**	**Associated *Mup* gene**	**1**	**2**	**3**	**4**	**5**	**6**	**7**	**8**	**9**	**10**	**11**	**12**	**13**	**14**	**15**
tr|P11588	MUP1	x								x	x	x	x	x	x	
tr|P11589	MUP2			x						x	x					
tr|P02762/P04938	MUP6/8/11	x		x				x	x	x	x	x		x	x	x
tr|B5X0G2	MUP17	x		x					x	x	x	x	x	x	x	
tr|Q5FW60	MUP20			x	x	x	x	x								

(B) POOLED SAMPLE

		Spot number																
**Uniprot number**	**Associated *Mup* gene**	**16**	**17**	**18**	**19**	**20**	**21**	**22**	**23**	**24**	**25**	**26**	**27**	**28**	**29**	**30**	**31**	**32**
tr|P11588	MUP1	x	x			x	x		x					x	x	x		
tr|P11589	MUP2		x	x	x				x				x	x	x	x		
tr|P02762/P04938	MUP6/8/11	x		x	x	x	x	x	x	x	x	x	x		x		x	x
tr|B5X0G2	MUP17			x		x				x	x	x				x	x	
tr|Q5FW60	MUP20				x	x												
tr|B0V388	MUP13			x											x			

Supplemental table S4. Comparison for MUP proteins detected in pairs of 2D-PAGE spots using iontrap (left column) and QTOF (right column) mass spectrometry. Labeling of spots as in Supplemental figure S3B and S5. Grey entries indicate proteins found by both MS methods.

	**Iontrap mass spectrometry**		**QTOF mass spectrometry**	
**Spot number**	**Uniprot number**	**Associated *Mup* gene**	**Uniprot number**	**Associated *Mup* gene**
**1**	tr|P11588	MUP1	tr|P11589	MUP2
	tr|P02762	MUP6/8/11	tr|Q3KQQ2	MUP3
	tr|P04938	MUP6/8/11	tr|A2ANT5	MUP4
	tr|B5X0G2	MUP17	tr|A2RSZ7	MUP5
			tr|A2AV72	MUP6
			tr|L7MUC7	MUP7
			tr|A2CEK6	MUP13
			tr|B8JI96	MUP14
			tr|B5TE76	MUP14/17
			tr|A9C497	MUP19
			tr|Q5FW60	MUP20
			tr|A9R9V7	MUP21
**6**	tr|Q5FW60	MUP20	tr|P11589	MUP2
			tr|Q3KQQ2	MUP3
			tr|A2ANT5	MUP4
			tr|Q58EV3	MUP7
			tr|P04938	MUP6/8/11
			tr|A2BIN1	MUP10
			tr|A9R9W0	MUP17
			tr|B5X0G2	MUP17
			tr|Q5FW60	MUP20
**7**	tr|P02762	MUP6/8/11	tr|A2AKN9	MUP2
	tr|P04938	MUP6/8/11	tr|A2ANT5	MUP4
	tr|Q5FW60	MUP20	tr|B5TE77	MUP5
			tr|A2RSZ7	MUP5
			tr|A2ANT6	MUP6
			tr|A2AV72	MUP6
			tr|P04938	MUP6/8/11
			tr|B5TE76	MUP14/17
			tr|A9R9W0	MUP17
			tr|B5X0G2	MUP17
			tr|Q5FW60	MUP20
**8**	tr|P02762	MUP6/8/11	tr|Q3KQQ2	MUP3
	tr|P04938	MUP6/8/11	tr|A2ANT5	MUP4
	tr|B5X0G2	MUP17	tr|A2RSZ7	MUP5
			tr|A2ANT6	MUP6
			tr|P04938	MUP6/8/11
			tr|B0V388	MUP13
			tr|A2CEK6	MUP13
			tr|B5TE76	MUP14/17
			tr|Q5FW60	MUP20
			tr|Q80Y18	MUP21
			tr|A9R9V7	MUP21

Supplemental table S4. continued

	Iontrap mass spectrometry		QTOF mass spectrometry	
**Spot number**	**Uniprot number**	**Associated *Mup* gene**	**Uniprot number**	**Associated *Mup* gene**
**9**	tr|P11588	MUP1	tr|B5TE77	MUP5
	tr|P11589	MUP2	tr|A2RSZ7	MUP5
	tr|P02762	MUP6/8/11	tr|A2ANT6	MUP6
	tr|P04938	MUP6/8/11	tr|A2AV72	MUP6
	tr|B5X0G2	MUP17	tr|A9R9W0	MUP17
			tr|B5TE76	MUP14/17
			tr|B5X0G2	MUP17
			tr|Q5FW60	MUP20
**10**	tr|P11588	MUP1	tr|P11589	MUP2
	tr|P11589	MUP2	tr|P04939	MUP3
	tr|P02762	MUP6/8/11	tr|Q3KQQ2	MUP3
	tr|P04938	MUP6/8/11	tr|A2ANT5	MUP4
	tr|B5X0G2	MUP17	tr|A2RSZ7	MUP5
			tr|A2AV72	MUP6
			tr|Q58EV3	MUP7
			tr|A2CEK6	MUP13
			tr|B5TE76	MUP14/17
			tr|Q5FW60	MUP20
**13**	tr|P11588	MUP1	tr|A2ANT5	MUP4
	tr|P02762	MUP6/8/11	tr|B5TE77	MUP5
	tr|P04938	MUP6/8/11	tr|A2RSZ7	MUP5
	tr|B5X0G2	MUP17	tr|A2ANT6	MUP6
			tr|A2AV72	MUP6
			tr|Q58EV3	MUP7
			tr|B5X0G2	MUP17
			tr|Q5FW60	MUP20
			tr|Q80Y18	MUP21
			tr|A9R9V7	MUP21

Supplemental table S5. Summary of MUPs detected in 2D-PAGE spots of the individual urine sample using QTOF mass spectrometry (Method C). Labeling of spots as in Supplemental figure S5.

		**Spot number**									
**Uniprot number**	**Associated *Mup* gene**	**1**	**6**	**7**	**8**	**9**	**10**	**13**	**33**	**34**	**35**
tr|L7MUC7	MUP7	x									
tr|B0V388	MUP13				x						
tr|B8JI96	MUP14	x								x	
tr|A9C497	MUP19	x								x	
tr|A2BIN1	MUP10		x						x		
tr|P04939	MUP3						x		x		
tr|A2CEK6	MUP13	x			x		x				
tr|A2AKN9	MUP2			x					x	x	
tr|P11589	MUP2	x	x				x				x
tr|Q58EV3	MUP7		x				x	x			x
tr|Q80Y18	MUP21				x			x		x	x
tr|Q3KQQ2	MUP3	x	x		x		x		x		
tr|A9R9V7	MUP21	x			x			x		x	x
tr|P04938	MUP8/11		x	x	x				x	x	
tr|A9R9W0	MUP17		x	x		x			x	x	
tr|B5TE77	MUP5			x		x		x		x	x
tr|A2ANT6	MUP6			x	x	x		x		x	
tr|A2ANT5	MUP4	x	x	x	x		x	x			
tr|B5TE76	MUP14/17	x		x	x	x	x		x	x	
tr|A2AV72	MUP6	x		x		x	x	x		x	x
tr|B510G2	MUP17		x	x		x		x	x	x	x
tr|A2RSZ7	MUP5	x		x	x	x	x	x		x	x
tr|Q5FW60	MUP20	x	x	x	x	x	x	x	x	x	x

Supplemental table S6. Summary of MUPs detected in 2D-PAGE spots of the pooled urine sample using QTOF mass spectrometry (Method C). Labeling of spots as in Supplemental figure 6B.

		Spot number																									
**Uniprot number**	**Associated *Mup* gene**	**37**	**38**	**39**	**40**	**41**	**42**	**43**	**44**	**45**	**46**	**47**	**48**	**49**	**50**	**51**	**52**	**53**	**54**	**55**	**56**	**57**	**58**	**59**	**60**	**61**	**62**
tr|P04938	MUP8/11											x															
tr|B5TE76	MUP14/17																					x					
tr|A2AKN9	MUP2																					x					
tr|B510G2	MUP17																					x					
tr|B0V388	MUP13																										x
tr|A2BIN1	MUP10		x																								
tr|A9R9W0	MUP17							x						x													
tr|Q5FW60	MUP20					x																	x				
tr|B8JI96	MUP14										x	x						x									
tr|Q58EV3	MUP7										x	x						x		x							
tr|A9C497	MUP19					x							x									x		x			
tr|P11589	MUP2	x				x									x		x										
tr|Q80Y18	MUP21														x				x	x							x
tr|L7MUC7	MUP7							x							x								x				x
tr|A2CEK7	MUP14				x							x			x	x	x		x								
tr|B5TE77	MUP5							x	x	x	x	x					x	x					x		x	x	x
tr|A2ANT6	MUP6	x	x	x		x	x			x			x		x	x		x			x						
tr|A2RSZ7	MUP5				x				x		x	x		x			x		x	x		x	x		x	x	x
tr|Q3KQQ2	MUP3	x	x	x	x	x	x	x		x			x	x								x	x				x
tr|A2ANT5	MUP4	x	x		x	x	x	x	x	x	x	x	x	x	x	x	x	x									x
tr|A2AV72	MUP6	x	x	x	x		x	x	x	x	x	x		x	x	x	x		x				x				x
tr|A2CEK6	MUP13	x	x	x	x	x	x	x	x	x	x			x		x			x	x	x	x	x	x			x
tr|A9R9V7	MUP21	x	x	x	x	x	x	x	x	x	x	x	x	x	x	x	x	x	x	x	x	x	x		x	x	

Supplemental table S7. Information on microsatellite markers

Marker group	Marker ID	Location	Forward primer	Reverse primer	Dye color	Annealing temperature (°C)
Distantly flanking MUP cluster	D4Nds6	chr4:52838656-52838895	5'-CGGGGAAGGTTGTTTGTTTG	5'-AGGCCAGCAATGTAGAAAGG	TAM	58
Distantly flanking MUP cluster	D4Mit288	chr4:56769379-56769494	5'-ACATTCAGCAAAGACTGAGCAC	5'-TGCCATTTGTTATAGACCATGC	HEX	58
Distantly flanking MUP cluster	D4Mit243	chr4:59529357-59529501	5'-AGCCCTACTGATTGCTCTCC	5'-TGGAAAGTTGAAAACCACTGC	TAM	56
Distantly flanking MUP cluster	D4Mit217	chr4:59833227-59833362	5'-ACTCAATTAGGTTGTTCAGATAGCC	5'-GGCACTTGCTGCCACATC	FAM	56
Distantly flanking MUP cluster	D4Mit164	chr4:59402240-59402381	5'-AACACATATATACCAAGGCAGCAC	5'-ATTTCCACCCTGTCCACTCC	FAM	64
Distantly flanking MUP cluster	D4Mit17	chr4:63365294-63365441	5'-GCCAACCTCTGTGCTTCC	5'-CCTCTGACATCCACACACATC	HEX	60
Distantly flanking MUP cluster	D4Mit139	chr4: 55250931-55251076	5'-TCAAACTGGGAAGAGCCAAG	5'-GCCGTAGAAGAGAAGTAATTTTTCC	TAM	58
Distantly flanking MUP cluster	D4Mit241	chr4: 55755707-55755925	5'-TTTCCAGTGTTGTCCAGAGC	5'-AAGGCAAATCACTAGGTGCTG	FAM	58
Closely flanking MUP cluster	MUP1	chr4:58954025-58954135	5´-CCGTGGTAAGTAGTGAGGCA	5´-ATGCATGAGACACTGGAGCC	HEX	64
Closely flanking MUP cluster	MUP2	chr4:59170558-59170818	5´-AAGGTCAAAGGGAGTGGGGG	5´-TGTGGAGTTTCTACCAGCCA	TAM	64
Closely flanking MUP cluster	MUP3	chr4:59349261-59349602	5´-TGCCCACTGAGCCATTTCAC	5´-AGTCCCTTCTGGGTGTTTGA	TAM	64
Closely flanking MUP cluster	MUP4	chr4:59558574-59558792	5´-TGTTCAACCTTGAGTGGGCA	5´-GAAGACCTAAGCCCCTGGTG	FAM	64
Closely flanking MUP cluster	MUP5	chr4:59759850-59759968	5´-TGTTTCCAAGTGGCCAAAGC	5´-GGCCTAGCTCTCTCAGGGAT	TAM	64
Closely flanking MUP cluster	MUP6	chr4:62365813-62366143	5´-CTGATCCCTAGCTGGAATGGTG	5´-CCTACTCCCTGCCCAGTCAA	FAM	64
Closely flanking MUP cluster	MUP7	chr4:62544555-62544762	5´-CCTGCAGGTCTACAAAGGGT	5´-GGTAGTGGGGAGTGGAACCT	HEX	64
Closely flanking MUP cluster	MUP8	chr4:62746101-62746256	5´-TGGCACACAATGTCACATGC	5´-AAAACCAGCATGCACAAGGG	FAM	64
Closely flanking MUP cluster	MUP9	chr4:62956825-62957023	5´-AGTCCACACAGCTCCTAAGC	5´-ACCGCATGTGGGAGTCATC	TAM	64
Closely flanking MUP cluster	MUP10	chr4:63160171-63160411	5´-GACTTCTGGTCTCCCCAAGC	5´-TCACAAGTTCCTTCTGGGGC	FAM	64
inside MUP cluster	MUP641	chr4:59955985-59956193	5'-TAGCATTGCCAAGCCTTTTC	5'-TGCCCAGCTTTTCATTTTTAG	HEX	56
inside MUP cluster	MUP10639	chr4:60475672-60476069	5'-GGTCCTCTGGAAGCATTGTC	5'-TTTTGTGATGTAGGCGCATC	HEX	56
inside MUP cluster	MUP85	chr4:61698769-61698916	5'-GGGAGTGGAGTGTAGGCAAC	5'-TCCCTCACATAACTTCTTGTGC	HEX	56
inside MUP cluster	MUP652	chr4:61833506-61833748	5'-ATCCTAATTGCCTGCCACAG	5'-TTGCTGACAAAACAGAAAAAGC	TAM	56
inside MUP cluster	MUP978	chr4:59964273-59964635	5'-TTGGCACTGGAATAAGAGACC	5'-TCTAGCATCTCCCTTTTGTGTG	FAM	64
inside MUP cluster	MUP58	chr4:60081418-60081542	5'-CCACAGCCATGCACAAATAG	5'-ATGGCTCCTTATGTTCCAGG	HEX	64
inside MUP cluster	MUP287	chr4:60438730-60438975	5'-TTGGTGACAATAGAGAAAAGGC	5'-CAGTGCTAGACACCAGGCAG	FAM	64
inside MUP cluster	MUP620	chr4:60626509-60626698	5'-GGTATAAAGCGAGTGATTGCC	5'-TCTCCAGCAGCCATGTAAAG	HEX	64
inside MUP cluster	MUP8046	chr4:61807297-61807473	5'-TGTGTGCATGCCTGTGTATG	5'-AGAACTGGGGAGAGAGAGGC	TAM	64
inside MUP cluster	MUP998	chr4:62150478-62150801	5'-TCAGTGAAACCAGAGGTCCC	5'-ACCTGAAAGACGTGGTCCTG	TAM	64

Supplemental table S7. continued

Marker group	Marker ID	Location	Forward primer	Reverse primer	Dye color	Annealing temperature (°C)
MHC-linked	D17Mit103	chr17: 34184468-34184614	5'-TACCACCTGGGCTACACCTC	5'-GCAATGCTTAGGTTAAAGCAGG	FAM	60
MHC-linked	D17Mit171	chr17: 34312441-34312579	5'-TTGGTATCTGCACTCAACTTGA	5'-TTATTTCATTTACTCGTGTGTGGG	HEX	60
MHC-linked	D17Mit13	chr17: 35079616-35079854	5'-TGCAGGCAAGATCCAAGAAG	5'-GAAAGAGGGTGTCGATGCTC	TAM	60
MHC-linked	D17Mit125	chr17: 36498280-36498471	5'-GGATTCCACAGGCATTGC	5'-TCCTGACTACCCCCAACTTG	TAM	60
MHC-linked	D17Mit24	chr17: 37482091-37482306	5'-ACCTCTCACCTCTCTCTGTG	5'-GCAAGTTTAGGGATCTTTTCTCC	HEX	60
MHC-linked	D17Saha1-4	chr17: 34314028-34314135	5'-CGACTGTAGAACCTTAGCCTG	5'-TGGAGCTGTCCTCCTTGTAG	TAM	57
MHC-linked	D17Mit21	chr17: 34243234-34243371	5'-TAACACCAGACATTGACCTC	5'-AGTCTAGATATGTGTCTCCC	FAM	56
MHC-linked	D17Nds3	chr17: 35204978-35205113	5'-TTCCTGTGGCGGCCTTATCAG	5'-AGACAATGGGTAACAGAGGCA	FAM	58
Other chromosomes	D2Mit380	chr2: 69617772-69617919	5'-CCTCAGGTCTGAAATGAGGTG	5'-AATGATGTGCATGTGCGC	FAM	57
Other chromosomes	D10Mit20	chr10: 67012408-67012633	5'-CACCCTCACACAGATATGCG	5'-GCATTGGGAAGTCCATGAGT	HEX	57
Other chromosomes	D9Mit135	chr9: 86648657-86648794	5'-ATTACATAGTCACTCTGAATG	5'-ACTTTTAGCAATTAGTAATTC	TAM	57
Other chromosomes	D7Mit227	chr7: 36580590-36580679	5'-GAGTCCTCAGCAGATATTACTCAGC	5'-CTGATGTCTCATCATTTGGGG	FAM	55
Other chromosomes	D5Mit25	chr5: 113883692-113883925	5'-AACACACCTCCATACTGGTCG	5'-GGCTAACTGAAATTGTTTTGTGC	HEX	55
Other chromosomes	D19Mit39	chr19: 29209335-29209590	5'-GGAGGTCTCAGGAAATATTACTCC	5'-ATTCCTGTGTAAAGGTGGATGG	HEX	55
Other chromosomes	D1Mit404	chr1: 177561852-177561968	5'-AGGAATAGAAAAATCAGCAAGCC	5'-CCATTGCCCTTGCTTTAGAA	TAM	55
Other chromosomes	D6Mit138	chr6: 4503823-4503937	5'-GCTCTTATTAATGAAGAAGAAGGAGG	5'-CAAAGAAAGCATTTCAAGACTGC	TAM	55
Other chromosomes	D9Mit34	chr9: 89748403-89748651	5'-AGTTTTGGCTAGTATAGGTT	5'-CATGGAACCTTAGAGCATGAG	FAM	55
